# Effect of a Web-Based Heartfulness Program on the Mental Well-Being, Biomarkers, and Gene Expression Profile of Health Care Students: Randomized Controlled Trial

**DOI:** 10.2196/65506

**Published:** 2024-12-16

**Authors:** Jayaram Thimmapuram, Kamlesh D Patel, Deepti Bhatt, Ajay Chauhan, Divya Madhusudhan, Kashyap K Bhatt, Snehal Deshpande, Urvi Budhbhatti, Chaitanya Joshi

**Affiliations:** 1 WellSpan Health York, PA United States; 2 Heartfulness Institute Hyderabad India; 3 Hospital for Mental Health Ahmedabad India; 4 Department of Graduate Medical Education Harvard Medical School Boston, MA United States; 5 I.I Pharmacy College New Vallabh Vidyanagar India; 6 Sneh Rehab and Education Center Mumbai India; 7 Gujarat Biotechnology Research Center Ahmedabad India

**Keywords:** heartfulness, meditation, stress, anxiety, depression, interleukins, gene expression, dehydroepiandrosterone, DHEA, gene, mental health, meditation, randomized study, web-based program, mental well-being, well-being, mental, health care students, student, mRNA, messenger ribonucleic acid, youth, young adults, web-based, biomarker, RNA, bioinformatics, randomized, statistical analysis, nursing, physiotherapy, pharmacy

## Abstract

**Background:**

Health care students often experience high levels of stress, anxiety, and mental health issues, making it crucial to address these challenges. Variations in stress levels may be associated with changes in dehydroepiandrosterone sulfate (DHEA-S) and interleukin-6 (IL-6) levels and gene expression. Meditative practices have demonstrated effectiveness in reducing stress and improving mental well-being.

**Objective:**

This study aims to assess the effects of Heartfulness meditation on mental well-being, DHEA-S, IL-6, and gene expression profile.

**Methods:**

The 78 enrolled participants were randomly assigned to the Heartfulness meditation (n=42, 54%) and control (n=36, 46%) groups. The participants completed the Perceived Stress Scale (PSS) and Depression Anxiety Stress Scale (DASS-21) at baseline and after week 12. Gene expression with messenger RNA sequencing and DHEA-S and IL-6 levels were also measured at baseline and the completion of the 12 weeks. Statistical analysis included descriptive statistics, paired *t* test, and 1-way ANOVA with Bonferroni correction.

**Results:**

The Heartfulness group exhibited a significant 17.35% reduction in PSS score (from mean 19.71, SD 5.09 to mean 16.29, SD 4.83; *P*<.001) compared to a nonsignificant 6% reduction in the control group (*P*=.31). DASS-21 scores decreased significantly by 27.14% in the Heartfulness group (from mean 21.15, SD 9.56 to mean 15.41, SD 7.87; *P*<.001) while it increased nonsignificantly by 17% in the control group (*P*=.04). For the DASS-21 subcomponents—the Heartfulness group showed a statistically significant 28.53% reduction in anxiety (*P*=.006) and 27.38% reduction in stress (*P*=.002) versus an insignificant 22% increase in anxiety (*P*=.02) and 6% increase in stress (*P*=.47) in the control group. Further, DHEA-S levels showed a significant 20.27% increase in the Heartfulness group (from mean 251.71, SD 80.98 to mean 302.74, SD 123.56; *P*=.002) compared to an insignificant 9% increase in the control group (from mean 285.33, SD 112.14 to mean 309.90, SD 136.90; *P*=.10). IL-6 levels showed a statistically significant difference in both the groups (from mean 4.93, SD 1.35 to mean 3.67, SD 1.0; 28.6%; *P*<.001 [Heartfulness group] and from mean 4.52, SD 1.40 to mean 2.72, SD 1.74; 40%; *P*<.001 [control group]). Notably, group comparison at 12 weeks revealed a significant difference in perceived stress, DASS-21 and its subcomponents, and IL-6 (all *P*<.05/4). The gene expression profile with messenger RNA sequencing identified 875 upregulated genes and 1539 downregulated genes in the Heartfulness group compared to baseline, and there were 292 upregulated genes and 1180 downregulated genes in the Heartfulness group compared to the control group after the intervention.

**Conclusions:**

Heartfulness practice was associated with decreased depression, anxiety, and stress scores and improved health measures in DHEA-S and IL-6 levels. The gene expression data point toward possible mechanisms of alleviation of symptoms of stress, anxiety and depression.

**Trial Registration:**

ISRCTN Registry ISRCTN82860715; https://doi.org/10.1186/ISRCTN82860715

## Introduction

Stress is the response to any demand where the internal resources may be challenged to meet the demand [1]. Mental health problems including stress, anxiety, and depression have increased over recent years, especially with the COVID-19 pandemic [2,3]. Health care students have a high degree of stress and burnout due to the number of hours spent on learning each week, the large body of clinical knowledge to master, and the challenges of balancing work and home life [4-6]. Stress and burnout are associated with negative consequences such as absenteeism, high turnover at the workplace, and decreased job satisfaction [7,8].

Despite the potentially serious consequences of stress and anxiety, there are few interventions designed to combat this problem in the student population. Educators need to develop an active awareness of mental well-being and should consider incorporating relevant instruction and interventions during the process of training health care professionals [9].

Well-being practices such as meditation are a potential tool and have been studied in health care settings [10-12]. Heartfulness meditation is a simple heart-based meditation system with gentle support from the trainers termed “yogic Transmission” aimed at attaining a balanced state of existence. The practices have been shown to reduce burnout, loneliness, and stress, and improve sleep [12-16]. In addition, changes in stress levels may correspond to changes in gene expression and interleukin-6 (IL-6) and dehydroepiandrosterone sulfate (DHEA-S) levels. IL-6 may participate in somatic maintenance efforts; hence, elevated levels may indicate that an organism is investing in the protection, preservation, and repair of somatic tissue [17-19]. DHEA-S is a hormone that counteracts cortisol and is considered an antiaging hormone. DHEA-S levels are relatively steady and reflect a cumulative change over time compared to cortisol levels that have diurnal fluctuations [20-22]. IL-6 levels have been studied in the context of well-being practices, such as meditation and in the setting of studying the effects of stress [18,19]. In practical settings, IL-6 is used as an inflammatory marker and may reflect the severity of infections [23]. Studies have assessed and demonstrated the effects of meditative practices on gene expression with a particular focus on stress-related inflammatory markers and associated biological pathways [17,24,25]. Stress, depression, and anxiety levels have been popularly measured with the Perceived Stress Scale (PSS) and Depression Anxiety Stress Scale (DASS-21) and have been validated in various settings [26,27].

This study investigated whether using a web-based Heartfulness meditation program with certified trainers is associated with improvements in mental well-being in health care professional students. This study also measured changes in the gene expression profile and DHEA-S and IL-6 levels.

## Methods

### Ethical Considerations

This study received approval from the Hospital for Mental Health Ethics Board, Ahmedabad (1568071-3). The study adhered to the guidelines for human research. The participants could opt out of the study at any time during the study period. The data were anonymized for analysis. No monetary compensation was offered to the participants. A written informed consent was obtained from all the participants.

### Overview

Participants were recruited by the institutional research team through internal communication media. The recruitment period was between February 17, 2022, and March 29, 2022. An introductory overview of the program was offered on the Zoom (Zoom Video Communications) platform and through follow-up announcements in the colleges. Eligible participants were students of nursing, physiotherapy, and pharmacy from 2 institutions in Gujarat, India. The study was registered with the ISRCTN Registry (ISRCTN82860715; date of registration: April 6, 2022). The study was conducted from April 2022 to July 2022.

### Enrollment and Randomization

A total of 78 eligible and willing participants enrolled in the study. Computer randomization was performed, with 42 (54%) participants randomly assigned to the intervention arm and 36 (46%) to the control arm. Based on the significance level (α=.05), an estimated effect size (*d*=0.80), and power (80%), the sample size estimated was 26 participants in each group. Considering factors of attrition, a higher number of participants were recruited. Volunteer response sampling was used after the study was announced through the educational institution’s internal communication.

All research participation was voluntary following an informed electronic consent. The eligibility criteria included participants aged 18 or older, able to read and understand the English language, and willing to participate in the study. All instructions were in English language and no translation was used for the questionnaires or the consent forms. Participants with any physical or mental conditions that would prevent them from sitting for the duration of meditation were excluded. Those participants who were able to provide data and blood samples both at baseline and at the follow-up after 12 weeks were included in the study. Those who failed to attend the final follow-up session for data collection and blood sample collection were excluded from the analysis. All participants were requested to fill out the PSS and DASS-21 forms at baseline before the start of the intervention. No other intervention was added during the study period. For any questions related to the study, the investigators were available throughout the study duration.

The intervention was web-based during the study period. The participants met with Heartfulness trainers via Zoom to guide them with the meditation sessions. The baseline and follow-up assessments were performed at week 0 and at week 12. All the participants (control group and Heartfulness group) were invited to a common location at week 0 and again at week 12 to fill out the questionnaires, and blood samples were collected at the same time.

### Blood Sample Collection

After proper identification of the participants and following aseptic precautions, 4.5 mL of blood sample was collected in a plain tube (red cap tube) for measuring IL-6 and DHEA levels. A blood sample of 2.5 mL was collected using a PAXgene RNA tube for RNA extraction and sequencing.

### Mental Well-Being Measures

#### Overview

This was a prospective randomized control trial assessing changes in the PSS and DASS-21 scores over a 12-week period. Both are validated questionnaires studied extensively in the literature [26,27].

#### PSS Questionnaire

The PSS consists of 10 items that measure the subjective feelings of the amount of stress in the participants by assessing thoughts and feelings in the previous month. Each question is scored from 0 (never) to 4 (very often) with a total possible score range of 0 to 40. A higher score indicates a high level of stress [26].

#### DASS-21 Questionnaire

The DASS-21 measures adverse mental states such as depression, anxiety, and stress in adults (patients and nonpatients). The 21 items on the questionnaire comprise a set of 3 self-reported scales designed to assess depression, anxiety, and stress [27].

### Biomarkers

For this study, DHEA-S and IL-6 markers were measured at baseline and at the 12-week follow-up period. DHEA-S and IL-6 levels were measured using chemiluminescence immunoassay, a rapid test involving a technique for determining sample concentrations based on the intensity of light emitted by a chemical and biological reaction.

### Gene Expression

#### Overview

To study the effect of Heartfulness meditation on stress and anxiety markers, we evaluated the alteration in gene expression. Blood samples from both the Heartfulness meditation group and the control group were collected at week 0 (baseline) and week 12 to study the changes in the expression profile due to meditation. RNA was extracted from the blood using the PAXgene Blood RNA kit (Cat# 762174) as per the manufacturer’s protocol. The quantitative and integrity analyses of RNA were done using the Qubit RNA BR Assay (Invitrogen, Cat# Q10211) and Tape Station using RNA screen tapes (Agilent, Cat# 5067–5576), respectively. After the confirmation, the quality control–passed samples were then processed through RNA sequencing.

#### Library Prep Protocol

The NEB Ultra II directional RNA-Seq Library Prep kit protocol was used to prepare libraries for messenger RNA (mRNA) sequencing (NEB, Cat# E7760L). An initial concentration of 500 ng of the total RNA was taken for the assay. The mRNA molecules were captured using magnetic Poly(T) beads (NEB, Cat# E7490L). Following purification, the enriched mRNA was fragmented using divalent cations under elevated temperatures. The cleaved RNA fragments were copied into first-strand complementary DNA (cDNA) using reverse transcriptase. Second strand cDNA synthesis was performed using DNA polymerase I and RNase H enzyme. The cDNA fragments were then subjected to a series of enzymatic steps that repair the ends, tails the 3’ end with a single “A” base, followed by ligation of the adapters. The adapter-ligated products were then purified and enriched using the thermal conditions of initial denaturation at 98 °C for 30 seconds, 12 cycles of –98 °C for 10 seconds, 65 °C for 75 seconds, and a final extension of 65 °C for 5 minutes. PCR products were then purified and checked for fragment size distribution on the Fragment Analyzer using HS NGS Fragment Kit (1-6000bp; Agilent, Cat# DNF-474-1000) or Tape Station using D1000 DNA Screen Tapes (Agilent, Cat# 5067-5582).

#### Sequencing Protocol

Prepared libraries were quantified using the Qubit HS Assay (Invitrogen, Cat# Q32854). The obtained libraries were pooled and diluted to the final optimal loading concentration. The pooled libraries were then loaded onto Illumina Novaseq 6000 to generate 150 bp paired-end reads or samples.

#### Bioinformatics Analysis

Using the FASTQC tool (version 0.11.9; Babraham Bioinformatics), a qualitative analysis of the sequenced data was carried out. Trimmomatic (version 0.36; Usadel lab) was used to trim low-quality reads, as well as Trueseq adapters. To make sure that the adapters and poor-quality reads were removed, the FASTQC procedure was repeated. Reads were confined to a minimum length of 100 bp. Using the HISAT2 (version 2.2.1) aligner, the trimmed paired fastq files were aligned to the GRCH37 genome (hg19). Gene annotation file and the genome fasta file were obtained from Ensembl. Using feature counts (version 2.0.6), the readings that were aligned to the exonic region were counted. The DESeq2 (version 1.40.2) R package’s differential expression analysis directly uses the count data produced by feature counts as an input. Genes with *P*-adjusted values (*P*=.05) and log2FC values >1.0 or 1.0 showed differential expression and were considered to be significant and further used for analysis. DESeq analysis was done between the pre-post Heartfulness group to gain insights on the bioprocesses significantly up and downregulated after the Heartfulness intervention. DESeq analysis between the post-Heartfulness (meditation and 12 weeks) and postcontrol (no meditation and 12 weeks) was done to gain insights on the bioprocesses significantly different between the 2 groups and to correlate the bioprocess with stress, anxiety and depression markers.

Apart from the enrichment analysis of upregulated and downregulated protein-coding genes, certain micro RNA (miRNA; precursor mi-RNA) reads were upregulated. The target genes of these miRNA were screened using miRwalk, mienturnet, Encori, and miEAA databases. The lists of genes present that were common in 2 or more databases were selected and compared to downregulated genes that were differentially expressed in this study.

#### Enrichment Analysis

Functional enrichment of differentially expressed genes was done using ClueGo (version 2.5.10) only for genes annotated as protein coding. Analysis was done using default parameters, selecting the network specificity option to detail, and enriching the pathways having the *P*-adj value<.05. Apart from that, functional enrichment analysis of the downregulated genes concerning micro-RNA (miRNA) was also studied using ClueGo.

#### Groupwise Expression Analysis Based on Participation

##### Overview

To assess the dose response effect to the meditation, we divided the Heartfulness group into 3 subgroups based on the number of Heartfulness sessions attended. Group 1, Group 2, and Group 3 were defined as having attended ≤33%, >33% to ≤60%, and >60% of the sessions, respectively. A total of 32 Heartfulness participants were divided into 9, 12, and 11 participants for groups 1, 2, and 3, respectively.

##### Kyoto Encyclopedia of Genes and Genomes Pathway Analysis

Kyoto Encyclopedia of Genes and Genomes Pathway analysis of genes involved in serotonergic, circadian entrainment, interleukin-17 pathway, and apoptosis was conducted.

##### Gene Ontology Enrichment Analysis

Functional enrichment of differentially expressed genes was performed using ClueGo (version 2.5.10) focusing only on genes annotated as protein-coding. The analysis was conducted using default parameters, selecting the network specificity option to “detail,” and enriching pathways with an adjusted *P* value ≤.05.

### Intervention

#### Heartfulness Meditation Group

All participants in the Heartfulness meditation group were invited for an orientation session via Zoom on the aspects of the study and the structure of the meditation protocol. Participants were also briefed about expectations during meditation sessions and were offered the contact details of trainers for scheduling meditation sessions.

During the guided meditation sessions, participants were walked through relaxation instructions by the trainers and were asked to gently place their attention on the source of light within their hearts. They were asked to simply tune into their hearts and be open to any experience they may have as opposed to trying to visualize the light. Participants were advised to gently redirect their attention toward their hearts if their attention drifted. The guided meditation sessions with the trainers were offered daily and lasted approximately 30 minutes. A total of 84 sessions were offered over a 12-week period. The number of sessions attended by the participants was logged by the volunteer trainers. No other interventions such as educational sessions were included during the study.

#### Control Group

An email was sent to the control group informing them about their cohort assignment. The control group did not receive any Heartfulness meditation-related instructions. They were instructed to carry on with their usual routine during the study period. They filled out the questionnaires and provided blood samples at the beginning and at the end of the study.

### Statistical Analysis

Descriptive statistics were used to examine participant demographics and work-related characteristics. Independent 2-tailed *t* test and Fisher exact test were used for baseline comparisons. The Levene test was performed to assess the homogeneity of variance. The 1-way ANOVA was used for assessing group differences. The level of significance was set at .05/4 following the Bonferroni correction, to account for multiple comparisons and potential type I errors. Paired *t* test analysis was conducted to evaluate within-group changes. The final analysis was done with the use of SPSS software (version 25.0; IBM Corp).

## Results

### Overview

**Figure 1:**
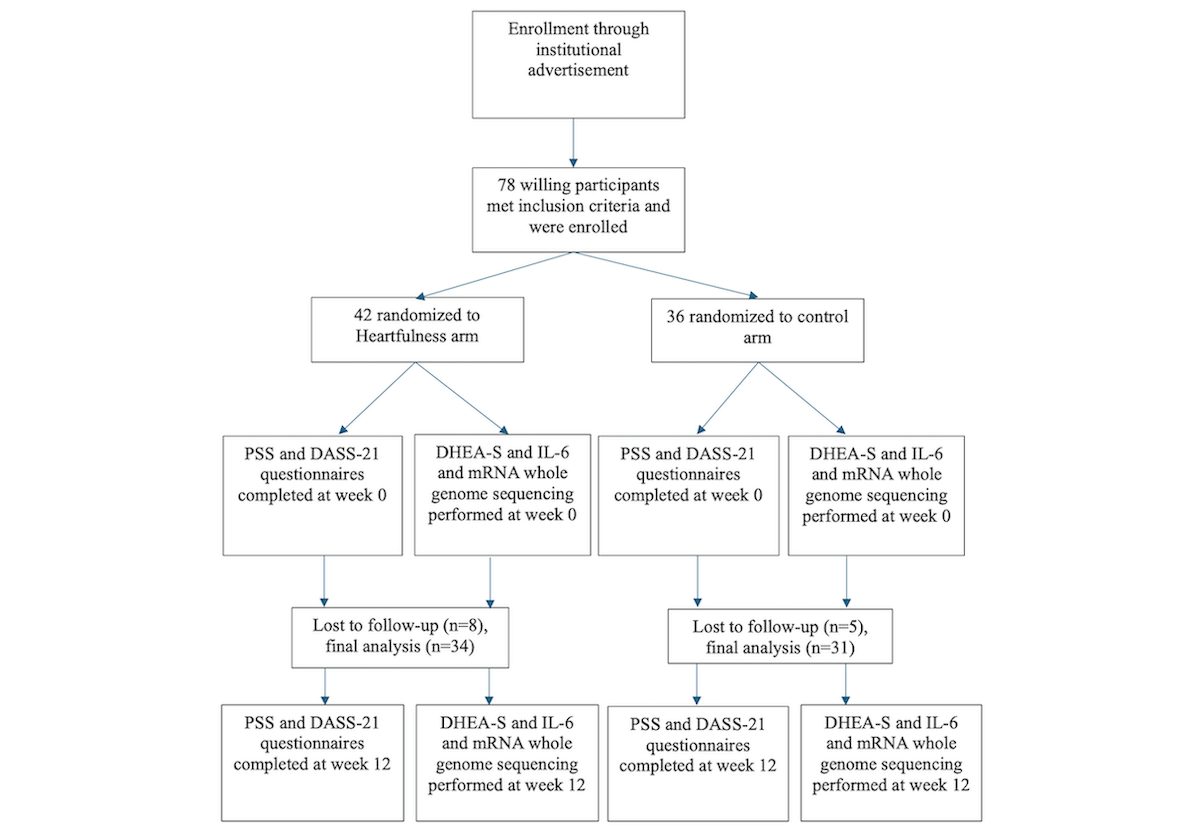
CONSORT flowchart. CONSORT: Consolidated Standards of Reporting Trials; DASS-21: Depression Anxiety Stress Scale; DHEA-S: dehydroepiandrosterone sulfate; IL-6: interleukin-6; mRNA: messenger RNA; PSS: Perceived Stress Scale.

A Consolidated Standards of Reporting Trials (CONSORT) flowchart outlines the enrollment of participants (Figure 1; Multimedia Appendix 1).

Participants’ demographic characteristics between Heartfulness and control groups at baseline are shown in Table 1. There were no statistically significant differences between the Heartfulness group and the control group at baseline.

**Table 1:** Demographics and characteristics.

Characteristic			Control group (n=31)		Heartfulness intervention group (n=34)		*t* test or Fisher exact test (*df*)	
**Age (years), mean (SD)**			20.55 (1.26)		20.15 (1.02)		0.161 (57.756)^a^	
**Sex, n (%)**								0.138^b^
	Female	25 (81)		32 (94)				
	Male	6 (19)		2 (6)				
**Perceived Stress Scale score, mean (SD)**			21.87 (4.65)		19.71 (5.09)		0.738 (63.000)^a^	
**Composite DASS-21^c^ score, mean (SD)**			22.42 (9.89)		21.15 (9.56)		0.932 (61.992)^a^	
**Depression subcomponent score of the DASS-21, mean (SD)**			6 (3.41)		5.35 (3.32)		0.833 (62.116)^a^	
**Anxiety subcomponent score of the DASS-21, mean (SD)**			7.77 (4.20)		6.59 (3.26)		0.724 (56.482)^a^	
**Stress subcomponent score of the DASS-21, mean (SD)**			8.65 (3.74)		8.91 (3.94)		0.945 (62.896)^a^	
**DHEA-S^d^ level, mean (SD)**			285.33 (112.15)		251.71 (80.99)		0.130 (54.177)^a^	
**IL-6^e^ level, mean (SD)**			4.52 (1.40)		4.93 (1.35)		0.737 (61.972)^a^	

^a^Independent 2-tailed *t* test.

^b^Fisher exact test.

^c^DASS-21: Depression Anxiety Stress Scale.

^d^DHEA-S: dehydroepiandrosterone sulfate.

^e^IL-6: interleukin-6.

At baseline, the Levene test confirmed the assumption of homogeneity of variance, and 1-way ANOVA indicated no difference in mean scores between the control group and the intervention group. This suggests that the groups were comparable, to begin with. At week 12, the Levene test confirmed homogeneity and there was a statistically significant difference in mean scores between the control group and the intervention group. The intervention group had a meaningful impact leading to improved outcomes compared to the control group at week 12. The homogeneity of variance is shown in Table 2.

**Table 2:** Homogeneity of variance.

	*F* test (*df*)	*P* value
PSS^a^ 1 (PSS scores at week 0)	0.113 (1, 63)	.74
PSS 2 (PSS scores at week 12)	1.215 (1, 63)	.27
DASS-21^b^ 1 (composite DASS-21 score at week 0)	0.007 (1, 63)	.93
DASS-21 2 (composite DASS-21 score at week 12)	0.806 (1, 63)	.37
D1 (depression subcomponent score of the DASS-21 at week 0)	0.045 (1, 63)	.83
D2 (depression subcomponent score of the DASS-21 at week 12)	1.565 (1, 63)	.22
A1 (anxiety subcomponent score of the DASS-21 at week 0)	0.126 (1, 63)	.72
A2 (anxiety subcomponent score of the DASS-21 at week 12)	1.018 (1, 63)	.32
S1 (stress subcomponent score of the DASS-21 at week 0)	0.005 (1, 63)	.94
S2 (stress subcomponent score of the DASS-21 at week 12)	0.025 (1, 63)	.88
DHEA-S^c^ 1 (DHEA-S blood levels at week 0)	2.358 (1, 63)	.13
DHEA-S 2 (DHEA-S blood levels at week 12)	0.560 (1, 63)	.46
IL-6 1^d^ (IL-6 blood levels at week 0)	0.114 (1, 63)	.74
IL-6 2 (IL-6 blood levels at week 12)	8.751 (1, 63)	.40

^a^PSS: Perceived Stress Scale.

^b^DASS-21: Depression Anxiety Stress Scale.

^c^DHEA-S: dehydroepiandrosterone sulfate.

^d^IL-6: interleukin-6.

The 1-way ANOVA was performed to assess the group differences. The results are shown in Table 3. The results have shown significant changes in PSS and DASS-21 scores along with the subcomponents of depression, anxiety, and stress scores from the DASS-21 questionnaire (all *P*<.05/4). In addition, IL-6 levels depicted a statistically significant difference at week 12 (*P*=.009), with no changes in DHEA-S.

**Table 3:** ANOVA results.

		Sum of squares	Mean square	*F* test (*df*)		*P* value	
**PSS^a^ 1 (PSS scores at week 0)**					3.187 (1, 63)		.18
	Between groups	76.011	76.011				
	Within groups	1502.543	23.850				
**PSS 2 (PSS scores at week 12)**					9.262 (1, 63)		.003^b^
	Between groups	297.947	297.947				
	Within groups	2026.607	32.168				
**DASS-21^c^ 1 (composite DASS-21 score at week 0)**					0.278 (1, 63)		.60
	Between groups	26.248	26.248				
	Within groups	5949.813	94.441				
**DASS-21 2 (composite DASS-21 score at week 12)**					24.155 (1, 63)		<.001^b^
	Between groups	1862.496	1862.496				
	Within groups	4857.719	77.107				
**D1 (depression subcomponent score of the DASS-21 at week 0)**					0.601 (1, 63)		.44
	Between groups	6.789	6.789				
	Within groups	711.765	11.298				
**D2 (depression subcomponent score of the DASS-21 at week 12)**					10.737 (1, 63)		.002^b^
	Between groups	141.178	141.178				
	Within groups	828.361	13.149				
**A1 (anxiety subcomponent score of the DASS-21 at week 0)**					1.633 (1, 63)		.21
	Between groups	22.807	22.807				
	Within groups	879.655	13.963				
**A2 (anxiety subcomponent score of the DASS-21 at week 12)**					34.459 (1, 63)		<.001^b^
	Between groups	370.184	370.184				
	Within groups	676.801	10.743				
**S1 (stress subcomponent score of the DASS-21 at week 0)**					0.078 (1, 63)		.78
	Between groups	1.153	1.153				
	Within groups	931.832	14.791				
**S2 (stress subcomponent score of the DASS-21 at week 12)**					11.523 (1, 63)		.001^b^
	Between groups	120.229	120.229				
	Within groups	657.309	10.433				
**DHEA-S^d^ 1 (DHEA-S levels at week 0)**					1.944 (1, 63)		.17
	Between groups	18325.652	18325.652				
	Within groups	593738.330	9424.418				
**DHEA-S 2 (DHEA-S levels at week 12)**					0.049 (1, 63)		.02
	Between groups	832.517	832.517				
	Within groups	1066172.701	16923.376				
**IL-6^e^ 1 (IL-6 levels at week 0)**					1.444 (1, 63)		.23
	Between groups	2.738	2.738				
	Within groups	119.486	1.897				
**IL-6 2 (IL-6 levels at week 12)**					7.366 (1, 63)		.009^b^
	Between groups	14.546	14.546				
	Within groups	124.409	1.975				

^a^PSS: Perceived Stress Scale.

^b^*P*<.05/4.

^c^DASS-21: Depression Anxiety Stress Scale.

^d^DHEA-S: dehydroepiandrosterone sulfate.

^e^IL-6: interleukin-6.

Further, paired *t* test analysis was conducted to evaluate within-group changes. The results are shown in Table 4. Paired *t* test analysis results showed a statistically significant reduction of PSS and DASS-21 scores within the Heartfulness group (all *P*<.05/4). The subcomponents of depression, anxiety, and stress levels from the DASS-21 questionnaire also showed a statistically significant reduction within the Heartfulness group at week 12 compared to baseline (all *P*<.05/4). In the control group, there was no statistically significant improvement in any of the scores (all *P*>.05/4). The effect size and percentage changes in mean values are reported in Table 4. Out of the 84 trainer-guided sessions, 20 participants attended >50% of the sessions, 8 of them attended >20% of the sessions, and the rest of them attended at least 4 sessions.

**Table 4:** Pre- and postintervention changes at baseline and end of study period.

Group and parameter			Pretest, mean (SD)	Posttest, mean (SD)		*t* test^a^ (*df*)		*P* value^b^		% change in mean values		Effect size (Cohen *d*)
**Intervention group**												
	Perceived Stress Scale score	19.71 (5.09)		16.29 (4.83)	3.661 (33)		<.001		–17.35%		0.628	
	Composite DASS-21^c^ score	21.15 (9.56)		15.41 (7.87)	3.679 (33)		<.001		–27.14%		0.631	
	Depression subcomponent score of the DASS-21	5.35 (3.32)		3.82 (3.19)	2.430 (33)		.02		–28.60%		0.417	
	Anxiety subcomponent score of the DASS-21	6.59 (3.25)		4.71 (2.97)	2.910 (33)		.006		–28.53%		0.499	
	Stress subcomponent score of the DASS-21	8.91 (3.94)		6.47 (3.10)	3.310 (33)		.002		–27.38%		0.568	
	DHEA-S^d^ levels	251.71 (80.98)		302.74 (123.56)	–3.317 (33)		.002		20.27%		0.369	
	IL-6^e^ levels	4.93 (1.35)		3.67 (1.00)	4.089 (33)		<.001		–25.56%		0.701	
**Control group**												
	Perceived Stress Scale score	21.87 (4.64)		20.58 (6.46)	1.040 (30)		.31		–6%		0.187	
	Composite DASS-21 score	22.42 (9.88)		26.13 (9.68)	–2.146 (30)		.04		17%		–0.385	
	Depression subcomponent score of the DASS-21	6.00 (3.40)		6.77 (4.04)	–1.219 (30)		.23		13%		–0.219	
	Anxiety subcomponent score of the DASS-21	7.77 (4.20)		9.48 (3.35)	–2.394 (30)		.02		22%		–0.430	
	Stress subcomponent score of the DASS-21	8.65 (3.73)		9.19 (3.36)	–0.724 (30)		.47		6%		–0.130	
	DHEA-S levels	285.33 (112.14)		309.90 (136.90)	–1.691 (30)		.10		9%		–0.304	
	IL-6 levels	4.52 (1.40)		2.72 (1.74)	4.179 (30)		<.001		–40%		0.750	

^a^Paired *t* test, confidence level 95%.

^b^*P*<.05/4 was considered statistically significant.

^c^DASS-21: Depression Anxiety Stress Scale.

^d^DHEA-S: dehydroepiandrosterone sulfate.

^e^IL-6: interleukin-6.

### Mental Well-Being Measures

#### PSS-10 Results

The PSS scores in the Heartfulness group showed a statistically significant reduction (from mean 19.71, SD 5.09 to mean 16.29, SD 4.83; *P*<.001) compared to the control group (from mean 21.87, SD 4.64 to mean 20.58, SD 6.46; *P*=.30). The percentage reduction of mean PSS scores in the Heartfulness group was 17.35% (Cohen *d*=0.628).

#### DASS-21 Results: Composite Score

The DASS-21 scores showed a statistically significant reduction in the Heartfulness group (from mean 21.15, SD 9.56 to mean 15.41, SD 7.87; *P*<.001). The percentage reduction of mean DASS-21 scores in the Heartfulness group was 27.14% (Cohen *d*=0.631). There was an insignificant increase in the DASS-21 scores in the control group (from mean 22.42, SD 9.88 to mean 26.13, SD 9.68; *P*=.04). The percentage increase of mean DASS-21 scores in the control group was 17% (Cohen *d*=–0.385).

#### DASS-21 Results: Subcomponent Scores

##### Depression

The depression subcomponent scores showed a 28.6% reduction in the Heartfulness group (from mean 5.35, SD 3.32 to mean 3.82, SD 3.19; *P*=.02), albeit not statistically significant. The control group showed a 13% increase in depression score, which was not statistically significant (from mean 6, SD 3.40 to mean 6.77, SD 4.04; *P*=.23). However, a significant but weak negative correlation was seen between the number of meditation sessions attended, with improvement in the depression subcomponent score with a correlation coefficient of –0.348 (*P*=0.044).

##### Anxiety

The anxiety subcomponent scores showed a statistically significant reduction in the Heartfulness group (from mean 6.59, SD 3.25 to mean 4.71, SD 2.97; *P*=.006). The percentage reduction in the mean anxiety scores was 28.53% (Cohen *d*=0.499). The anxiety subcomponent scores showed a 22% increase in the control group (from mean 7.77, SD 4.2 to mean 9.48, SD 3.35; *P*=.02), which was statistically insignificant.

##### Stress

The stress subcomponent scores showed a statistically significant reduction in the Heartfulness group (from mean 8.91, SD 3.94 to mean 6.47, SD 3.10; *P*=.002). The percentage reduction in the mean stress scores was 27.38% (Cohen *d*=0.568). The control group did not show any statistically significant change in the stress subcomponent scores (from mean 8.65, SD 3.73 to mean 9.19, SD 3.36; *P*=.47), with a 6% increase.

### Biomarkers

#### DHEA-S Levels

DHEA-S levels showed a statistically significant increase in the Heartfulness group (from mean 251.71, SD 80.98 to mean 302.74, SD 123.56; *P*=.002; Cohen *d*=0.369), with a percentage increase in the mean DHEA-S levels of 20.27% compared to the 9% in control group, which showed no statistically significant changes (from mean 285.33, SD 112.14 to mean 309.90, SD 136.90; *P*=.10).

#### IL-6 Levels

IL-6 levels showed a statistically significant decrease in both the groups: a 28.6% decrease in the Heartfulness group and a 40% decrease in the control group—(from mean 4.93, SD 1.35 to mean 3.67, SD 1.0; *P*<.001; Cohen *d*=0.701 [Heartfulness group] and from mean 4.52, SD 1.40 to mean 2.72, SD 1.74; *P*<.001; Cohen *d*=0.75 [control group]).

### Gene Expression Profile Results

#### Overview

**Figure 2:**
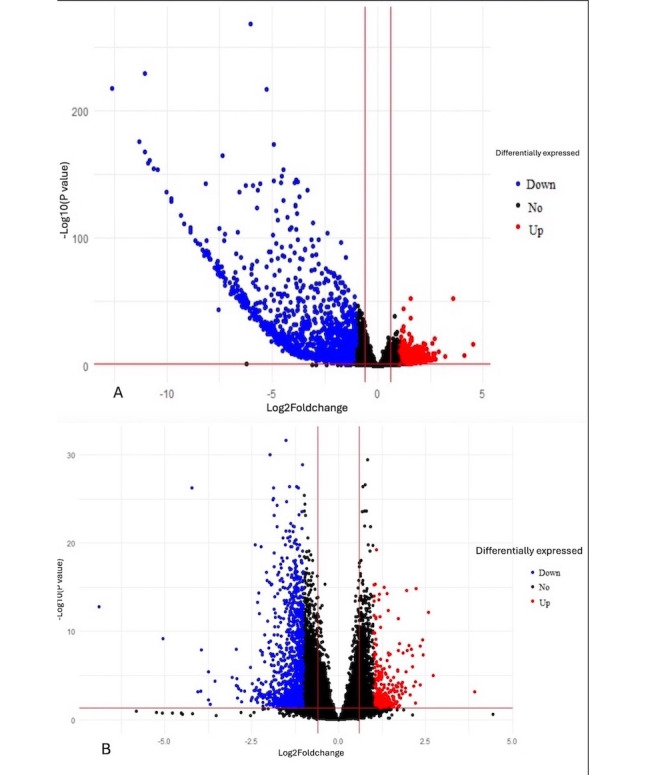
(A) Scatter plot showing genes differentially expressed in the pre-post Heartfulness group comparison. Red and blue dots indicate statistically significant upregulated and downregulated genes. Black dots represent a statistically insignificant differential expression of genes. (B) Scatter plot showing genes differentially expressed in the post-Heartfulness–postcontrol group comparison. Red and blue dots indicate statistically significant upregulated and downregulated genes. Black dots represent a statistically insignificant differential expression of genes.

**Figure 3:**
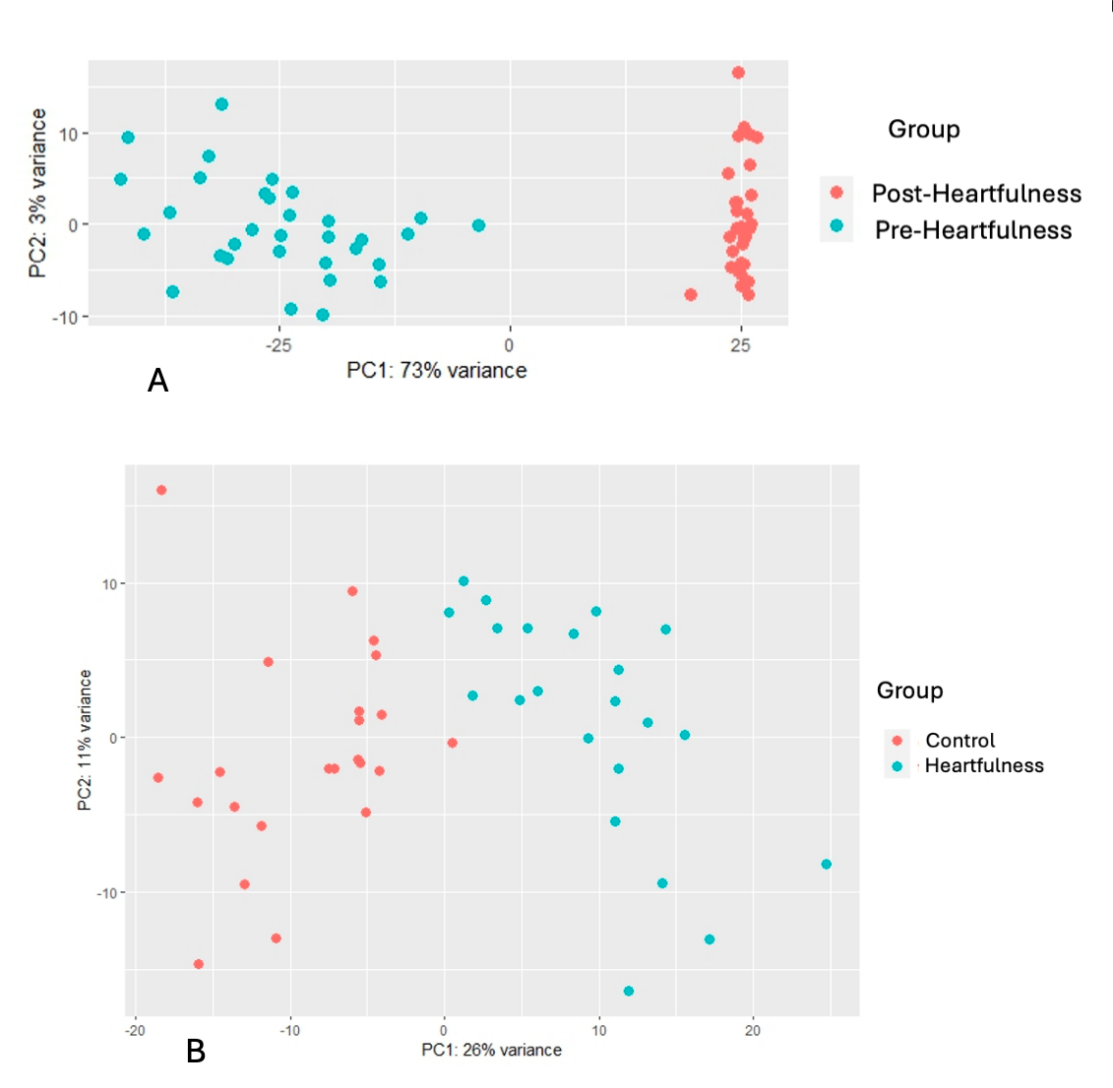
(A) PCA plot of pre- and post-Heartfulness DESeq data, clustering 2 groups separately. The graphical representation of the transcriptome profile includes pre- (0-week; cyan) and post-Heartfulness (12-week; orange) participants. Principal component 1 (PC1) and principal component 2 (PC2) account for 73% (x-axis) and 3% (y-axis) of the variance respectively in expression data. (B) PCA plot of post-Heartfulness and postcontrol DESeq data clustering after removing the outliers. The graphical representation of transcriptome profile of 22 post-Heartfulness (cyan) and 23 postcontrol (12-week; orange) participants, respectively. PC1 and PC2 account for 26% (x-axis) and 11% (y-axis) of the variance respectively in expression data. PCA: principal component analysis.

The percentage of reads aligned with the reference genome for mapping was more than 90% in all samples. Figure 2A-B depicts scatter plots illustrating differentially expressed genes in the pre-post Heartfulness group comparison and post-Heartfulness–postcontrol group comparison, respectively. The pre- and post-Heartfulness groups are shown in a principal component analysis plot cluster (Figure 3A), displaying the effective segregation of the groups. The principal component analysis plot, with outliers removed, is shown in Figure 3B for the 12-week post-Heartfulness and 12-week postcontrol groups. After the removal of the outliers, 23 postcontrol and 22 post-Heartfulness samples were incorporated for group comparisons. A total of 875 genes were identified to be upregulated, whereas 1539 genes were found to be downregulated in the interventional Heartfulness group compared to the baseline premeditation samples (week 0). A total of 292 genes were found to be upregulated and 1180 downregulated in the post-Heartfulness group compared to the postcontrol group. Overall changes in the bioprocesses are shown in Textboxes 1 and 2.

**Bioprocesses significantly enriched in the pre-post Heartfulness group comparison.**

**Upregulated bioprocesses**

- Histone modification involving acetyation
- Cell adherens junctions
- L dopamine and acetylecholine catabolic process
- Nicotinamide adenine dinucleotide (NAD) metabolic processes
- Mevalonate and farnesyl pathways
- Neuromuscular synaptic transmission
- Hydrogen peroxide mediated cell death process
- Immune cell receptor signaling pathways

**Downregulated bioprocesses**

- Cell differentiation pathways
- Cell-cell adhesion
- Catagen bioprocesses
- Hematopoietic bioprocesses
- Neuromuscular synaptic assembly and activity
- Tau protein kinase activity
- Protein assembly and Histon H3T3 kinase activity
- Apoptotic processes
- Vasopressin and prosaposin signaling

**Bioprocesses significantly enriched in the post-Heartfulness–postcontrol group comparison.**

**Upregulated bioprocesses**

- White blood cell and red blood cell differentiation
- Microtubule organization
- Glutathione transport
- Synaptic transmission
- Cell polarity bioprocesses
- Protein post translation modifications

**Downregulated bioprocesses**

- Lymphoid progenitor differentiation
- Protein acetylation
- Metallo-endopeptidase activity
- Golgi reassembly
- Fatty acid transport to peroxisomes

#### Functional Enrichment

##### Overview

Gene Ontology (GO) enrichment analysis was used to identify the contribution of genes in biological processes, enabling the identification of the pathway in which genes play a part.

##### GO Analysis of Pre-Post Heartfulness Group Comparison

**Figure 4:**
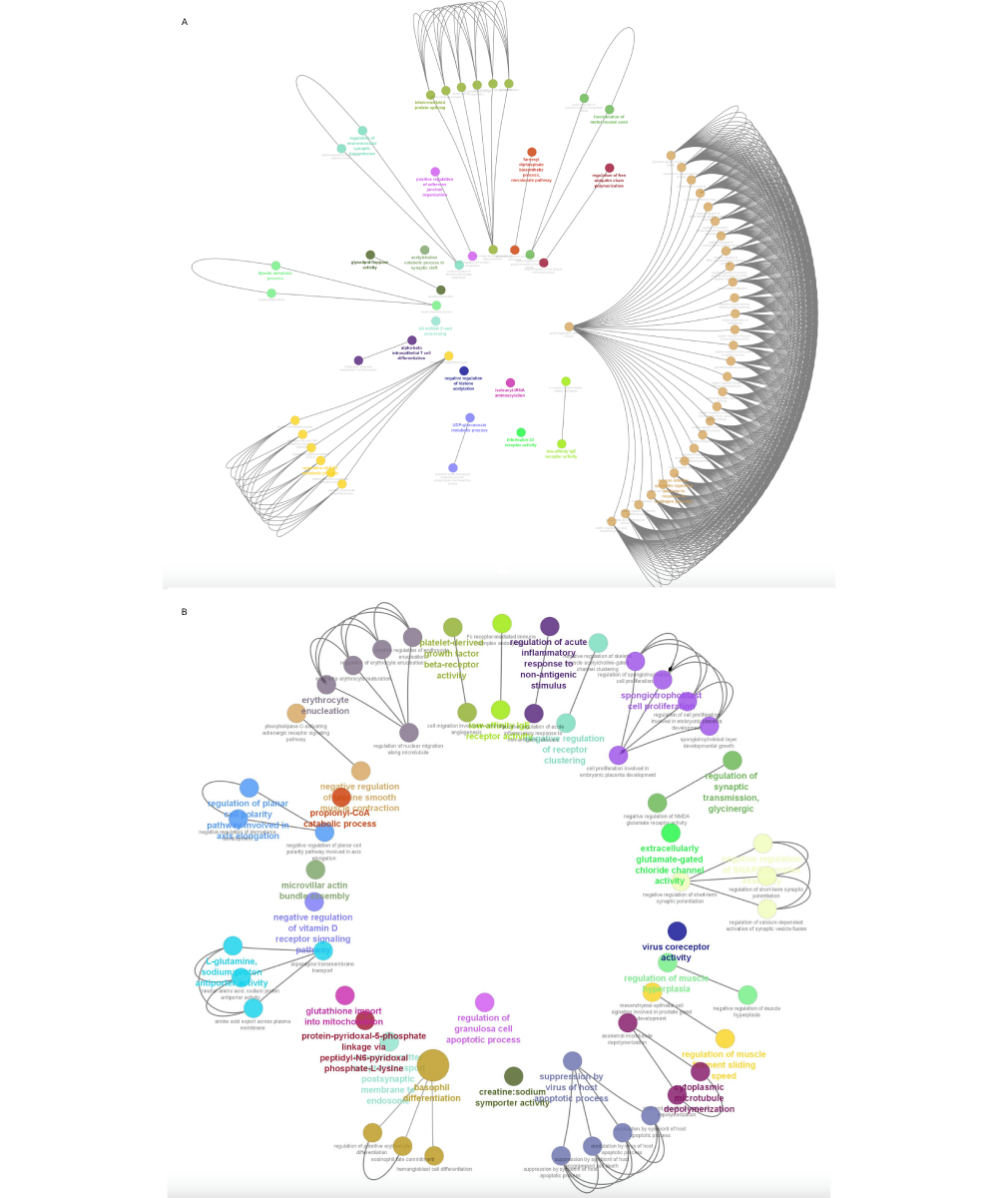
(A) Graphical representation of the enriched GO processes significantly upregulated in the pre-post Heartfulness group comparison. (B) Graphical representation of enriched GO processes significantly upregulated in post-Heartfulness–postcontrol group comparison. GO: Gene Ontology. Higher resolution images are available in Multimedia Appendices 3 and 4.

**Figure 5:**
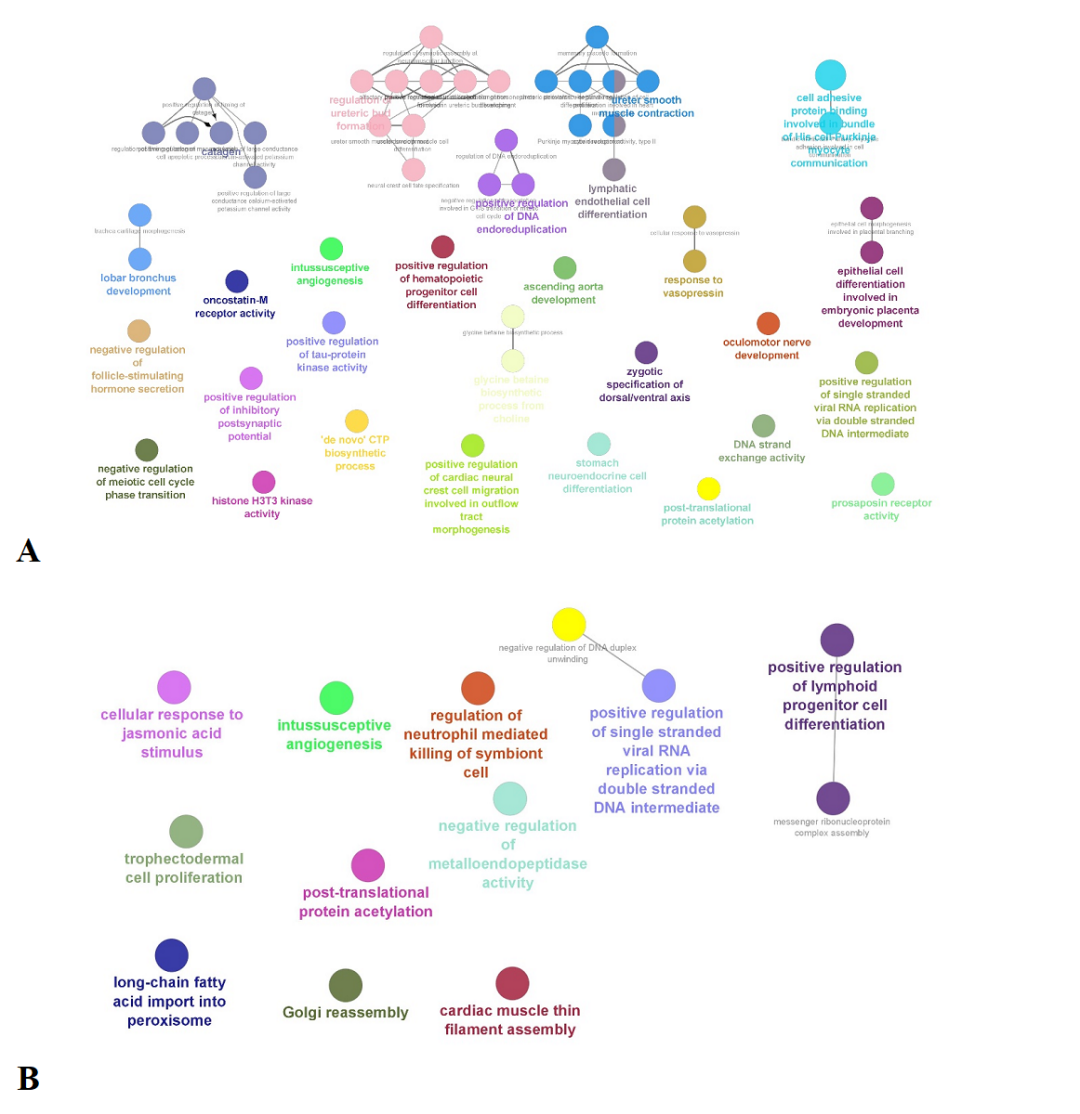
(A) Graphical representation of the enriched GO processes significantly downregulated in the pre-post Heartfulness group comparison. (B) Graphical representation of enriched GO processes significantly downregulated in the post-Heartfulness–postcontrol group comparison. GO: Gene Ontology.

The GO analysis of the upregulated protein-coding genes using ClueGo enriched biological processes showed the genes that were involved in histone acetylation, adherens junction, acetylcholine catabolic process, nicotinamide adenine dinucleotide metabolic processes, glucuronate and lipoate processes, immune cell receptor and differentiation pathway, farnesyl and mevalonate pathways, ubiquitin polymerization, L-dopamine processing, cell death-related processes in response to hydrogen peroxide, and neuromuscular synaptic transmission processes. Enriched upregulated pathways in the pre-post Heartfulness group are shown in Figure 4A. Downregulated protein-coding genes enriched biological processes are shown in Figure 5A involving cell adhesion protein involved in His-Purkinje myocytes, lymphatic endothelial differentiation, regulation of catagen process, synaptic assembly at neuromuscular junctions, processes involved in synaptic activity, tau-protein kinase activity, hematopoietic processes, prosaposin, vasopressin response, apoptotic process, histone H3T3 kinase activity, and protein acetylation processes. While additional developmental biology processes were enriched in the downregulated genes, we focused on the major processes that are relevant to our study objectives.

##### GO Analysis of Post-Heartfulness–Postcontrol Group Comparison

GO analysis of upregulated genes for the biological process were significantly enriched for the processes, such as transport of basophil and erythrocyte differentiation and enucleation process respectively, bioprocesses involving in the microtubule dynamics, membrane receptor organization, glutathione transport, synaptic transmission, planar cell polarity, membrane transport protein, and protein post translational modification. Upregulated bioprocesses are depicted in Figure 4B. Downregulated genes were mainly enriched in bioprocesses involved in the fatty acid import to peroxisome, Golgi reassembly, lymphoid progenitor cell differentiation, protein acetylation, and metalloendopeptidase activity. Downregulated bioprocesses are shown in Figure 5B.

#### Enriched Biological Processes

**Figure 6:**
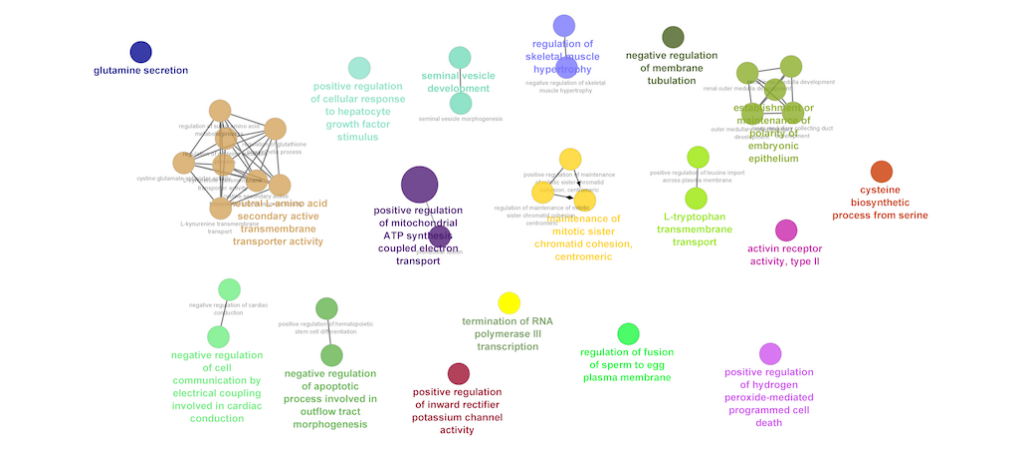
Biological processes enriched with miRNA-targeted genes. miRNA: micro RNA.

A total of 1285 target genes were found for the 17 upregulated miRNAs in the pre-post meditation group comparison. From these genes, we found 85 genes mapped to our list of downregulated genes. The GO biological process enrichment analysis indicated that these genes were involved in L-amino acid transmembrane transporter activity, hydrogen peroxide mediated cell death, L-tryptophan transport, glutamine secretion, involved in cardiac conduction, adenosine triphosphate coupled electron transport, and RNA pol III transcription-like processes (Figure 6).

The effect of Heartfulness meditation practices on circadian rhythm entrainment genes and the subgroup analysis according to the degree of participation in meditation sessions are reported in Multimedia Appendix 2.

## Discussion

### Principal Findings

This study shows an improvement in mental well-being with Heartfulness meditation offered with guidance by certified trainers. Stress, anxiety, and depression levels showed a decrease with 12 weeks of practice. DHEA-S levels showed a significant increase in the meditation group at the end of the study compared to the baseline scores. IL-6 levels decreased in both groups. Gene expression profiling with mRNA sequencing showed features suggestive of promoting central nervous system (CNS) homeostasis, neuroinflammatory protective effect, and reduction of oxidative stress among the meditators. The study findings support the positive effect of meditative practices on well-being.

Previous studies involving Heartfulness practices have shown improvements in mental well-being including improvement in burnout, loneliness, and stress along with an improvement in sleep in various study populations [12-16]. Compared to the previous studies, this study involved the practice of Heartfulness meditation exclusively in the health care student population and has shown a decrease in depression, anxiety, and stress scales. This study is in line with previous interventions that showed an improvement in mental well-being in clinical settings [11,17]. The measurement of biomarkers and underlying gene expression changes is a novel addition to the study of the effects of Heartfulness practices.

This study showed a significant elevation in the DHEA-S within the group with Heartfulness meditation practice. The DHEA-S, secreted by the adrenal cortex, may have an anti-cortisol effect and potentially cardioprotective, antidiabetic, antiobesity, and immunoenhancing properties, and may be involved in neural glial cell regulation and neuroplasticity [28-30]. DHEA-S declines with advancing age and is reported as an antiaging or youth hormone [22]. The levels are relatively steady, without much influence by the circadian rhythm, making DHEA-S a good biomarker for measuring cumulative changes in stress levels and overall health. Previous studies have shown an increase in DHEA-S levels with meditative practices and restful states of the brain [31-33]. It is likely that the practices of Heartfulness offer a restful state of the brain leading to an increase in the DHEA-S levels. This study adds to the body of literature favoring meditative practices in increasing the levels of DHEA-S levels.

IL-6 is an inflammatory marker that has been studied in the context of meditation practices. The results have been mixed. Moreover, this marker has been used more often in clinical settings and not in the general population. It can fluctuate with infections and thus the effect of practices such as meditation maybe minimal [23,34]. This might explain the findings of this study that showed decreased levels of IL-6 in both groups, indicating that other factors may have contributed to the changes observed. An increased participation in meditation showed a correlation with a reduction in the depression component.

Gene expression profiling suggested that meditation promoted CNS homeostasis, reduced neuroinflammation, and oxidative stress. This aligns with previous studies linking meditation to improved mental health [17,24]. The duration of this study is also in line with previous studies ranging from 8 to 12 weeks [12,13,17]. The study observed upregulation of pathways linked to neuroprotection, including picolinic acid biosynthesis (against neurotoxicity) and lipoate synthesis (antioxidant and neurotransmitter regulation). Additionally, reduced fatty acid oxidation and upregulation of mevalonate pathways suggest the potential for stress and depression alleviation [35-38].

Poor blood-brain barrier function has been related to stress and depression symptoms and upregulation of adherens junction organization in this study suggests meditation may improve blood-brain barrier function, potentially improving mental function [39]. Meditation downregulated the catagen process, a stress-related pathway [40]. Additionally, downregulation of the tryptophan transporter suggests reduced activity in the kynurenic acid pathway, potentially leading to increased serotonin synthesis and improved mood [41,42]. Increased glutathione transport to the mitochondria suggests reduced oxidative stress after meditation. This aligns with the downregulation of fatty acid import to the peroxisome, a primary site for fatty acid oxidation [43]. The study also observed the downregulation of pathways related to glutamate signaling, potentially reducing N-methyl-D-aspartate receptor activity and cholinergic levels, which have been linked to depression [44]. The genes related to circadian rhythm regulation have been linked to depression and were found to be altered with the practice [45]. These changes could be associated with the improvement of depression symptoms.

The subgroup analysis based on the participation rates in the meditation sessions revealed that higher participation in the meditation sessions showed a downregulation of inflammatory pathways compared to those with lesser participation. Most of these pathways were involved in immune signaling. Interleukin-4 (IL-4) upregulation has been linked to major depressive disorder and myeloid differentiation factor 88 is associated with neuroinflammation [46,47]. This downregulation included bioprocesses involved with inflammatory pathways and inflammatory response during wound healing. Another pathway involved with both apoptosis and inflammation, the Fas signaling pathway, was found to be downregulated [48].

There was downregulation of histone trimethyl transferase activity with brain-derived neurotrophic factor signaling with higher participation in the meditation sessions. This aligns with previous reports suggesting that hypermethylation of brain-derived neurotrophic factors is significantly associated with depression [49].

Pathways related to tau protein kinase activity, amyloid fibril formation, and several developmental processes were downregulated in the Heartfulness group potentially indicating involvement with the neurotoxin removal process [50]. These results point toward a positive effect on brain health with Heartfulness practices.

Furthermore, chronic stress is linked to neuroinflammation and increased cell death [51]. The downregulation of the interleukin-17 pathway, a key player in inflammation [52], could be a potential link between meditation and reduced inflammation.

The receptors 5HT2A and HTR7 have been linked to anxiety and depression and medications blocking these receptors act as antidepressants [53]. Interestingly, with higher meditation participation, there was a downregulation of HTR7 receptors, along with the MAOA gene. The MAOA gene has been reported to help break down neurotransmitters such as serotonin and norepinephrine. Research suggests that MAOA expression is increased in depression and inhibiting it can help restore healthy neurotransmitter levels and even protect neurons from cell death [54]. The downregulation of both MAOA and specific serotonin receptor genes (HTR7) with higher meditation participation could explain an alternative regulation of these pathways. Interestingly, this could also explain the negative correlation of depression scores with a higher number of meditation sessions attended.

To our knowledge, this is the first study in health care professional students including nursing, physiotherapy, and pharmacy assessing mental well-being, biomarkers, and gene expression. The participants had no formal meditation experience and were novice meditators indicating that this practice could be of practical use in real-world settings.

The Heartfulness meditation program used in the study demonstrates the potential to improve depression, stress, and anxiety levels, along with improving levels of DHEA-S and effecting gene expression. These results might be applicable to other student populations. Instituting similar programs more widely might benefit health care professionals.

### Limitations

The control group was a no-treatment group, with no alternative activity offered. Many factors influence inflammatory markers and, therefore, it is difficult to assess the exclusive effect of meditation practices. Participants who were unable to attend the study site on the day of completion could not be included in the analysis. There could also be many unknown sources of bias, including diet and health conditions along with the personal life factors of participants. Also, the long-term follow-up data on the lasting effects of Heartfulness meditation are lacking in this study.

### Conclusions

Heartfulness meditation practice appears to provide an improvement in mental well-being with reduced stress, anxiety, and depression levels in the health care professional student population. The web-based nature of this program shows this model to be applicable to the student lifestyle. Increased DHEA-S levels within the Heartfulness group and gene expression changes indicate possible underlying physiological processes associated with improved mental well-being changes. Gene expression profiling with mRNA sequencing suggested the promotion of CNS homeostasis, neuroinflammatory protective effect, antidepressant activity, and reduction of oxidative stress. Further research with a longer follow-up period of up to a year or more is recommended to evaluate the long-term effects of Heartfulness meditation on mental well-being and changes in the biomarkers.

## Appendix

Multimedia Appendix 1 CONSORT-EHEALTH (V 1.6.1) checklist

Multimedia Appendix 2 Effect on circadian rhythm entrainment genes and dose-response effect to the meditation sessions.

Multimedia Appendix 3 Higher resolution image of Figure 4A: Graphical representation of the enriched GO processes significantly upregulated in the pre-post Heartfulness group comparison.

Multimedia Appendix 4 Higher resolution image of Figure 4B: Graphical representation of enriched GO processes significantly upregulated in post-Heartfulness–postcontrol group comparison. GO: Gene Ontology.

## Abbreviations

cDNA: complementary DNA
CNS: central nervous system
CONSORT: Consolidated Standards of Reporting Trials
DASS-21: Depression Anxiety Stress Scale
DHEA-S: dehydroepiandrosterone sulfate
GO: Gene Ontology
IL-4: interleukin-4
IL-6: interleukin-6
miRNA: micro RNA
mRNA: messenger RNA
PSS: Perceived Stress Scale
