# Supplementary material for: Effect of a Web-Based Heartfulness Program on the Mental Well-Being, Biomarkers, and Gene Expression Profile of Health Care Students: Randomized Controlled Trial
Source: JMIR Bioinform Biotechnol. 2024 Dec 16;5:e65506. doi: 10.2196/65506 (PMC11686021; doi:10.2196/65506)
Supplement: Multimedia Appendix 2 [file bioinform_v5i1e65506_app2.docx]

**Downregulation of genes involved in the sleep regulation:**

In addition to examining the bioprocesses that underwent substantial changes because of meditation, we also examined the expression of genes involved in sleep as stress and sleep are closely correlated. We extracted the gene list involved in the circadian entrainment and serotonergic signaling from KEGG database. In this work, we discovered 12 genes in the pre- -post-Heartfulness group and 5 genes in the post-Heartfulness post-control group that have been linked to roles in the serotonergic and circadian rhythm pathways. In both comparisons, there was a downregulation of all these genes. The five genes identified in the post-Heartfulness post-control groups were specifically enriched for the serotonergic pathway, whereas the genes identified in the pre-post Heartfulness comparison were specifically enriched for the cholinergic (8/12), serotonergic (7/12), and circadian entrainment (10/12) pathways according to the stringDB gene interaction analysis (Supplementary Figures 1a-d).


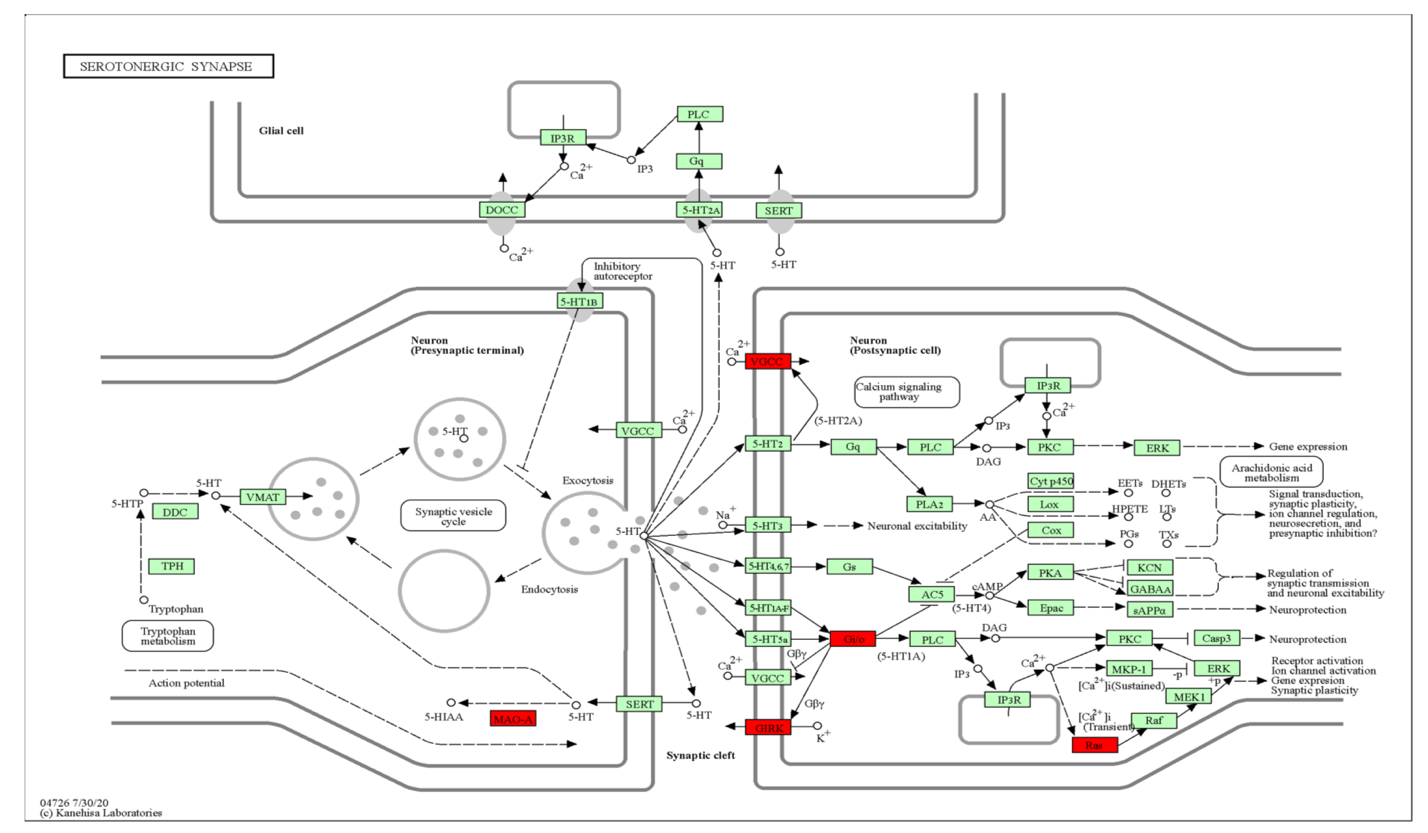


**Figure S1A: Genes (red) down regulated in the serotonergic pathways, in the pre-post-Heartfulness group**

**
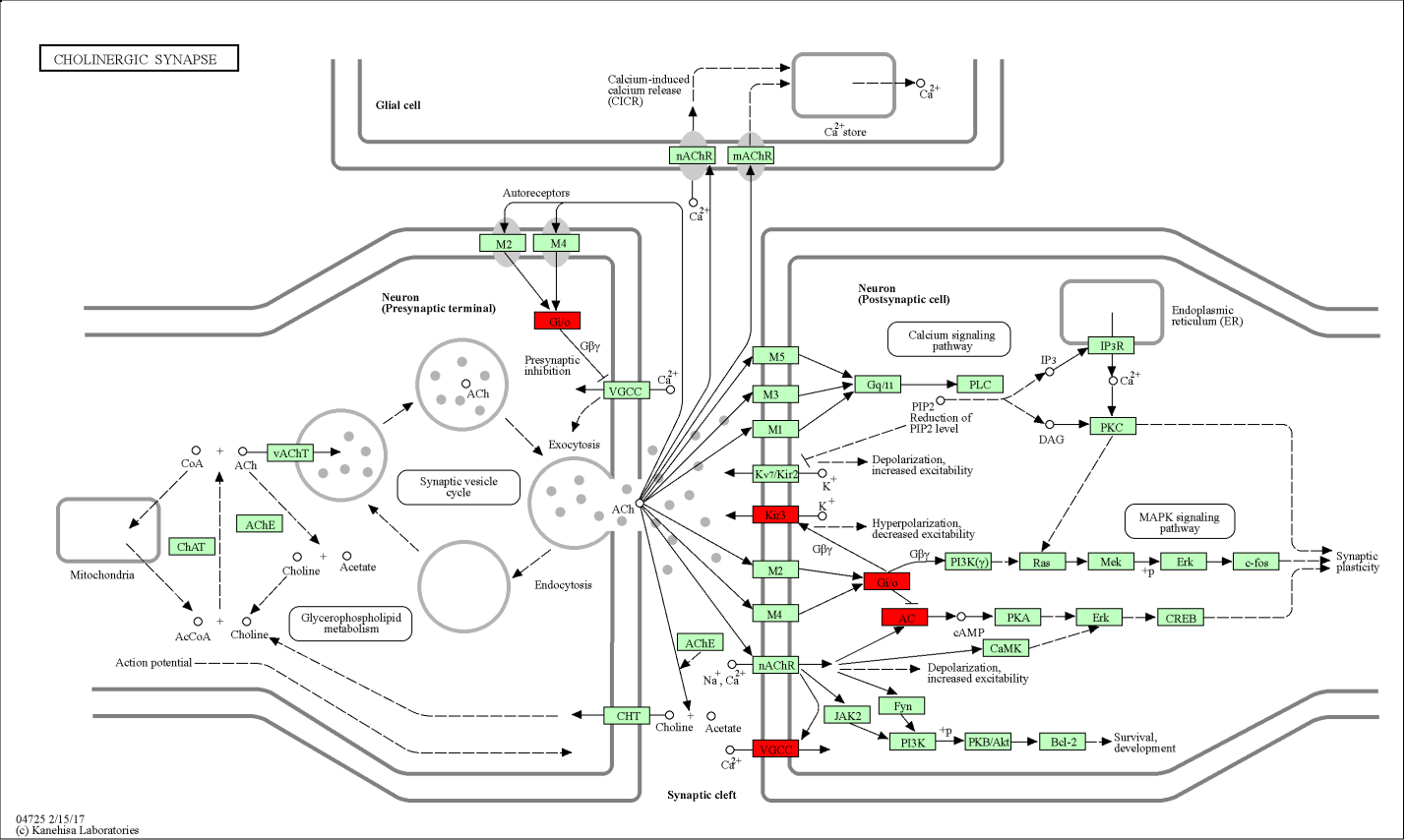
**

**Figure S1B: Genes (red) down regulated in the cholinergic pathways, in the pre-post-Heartfulness group**

**
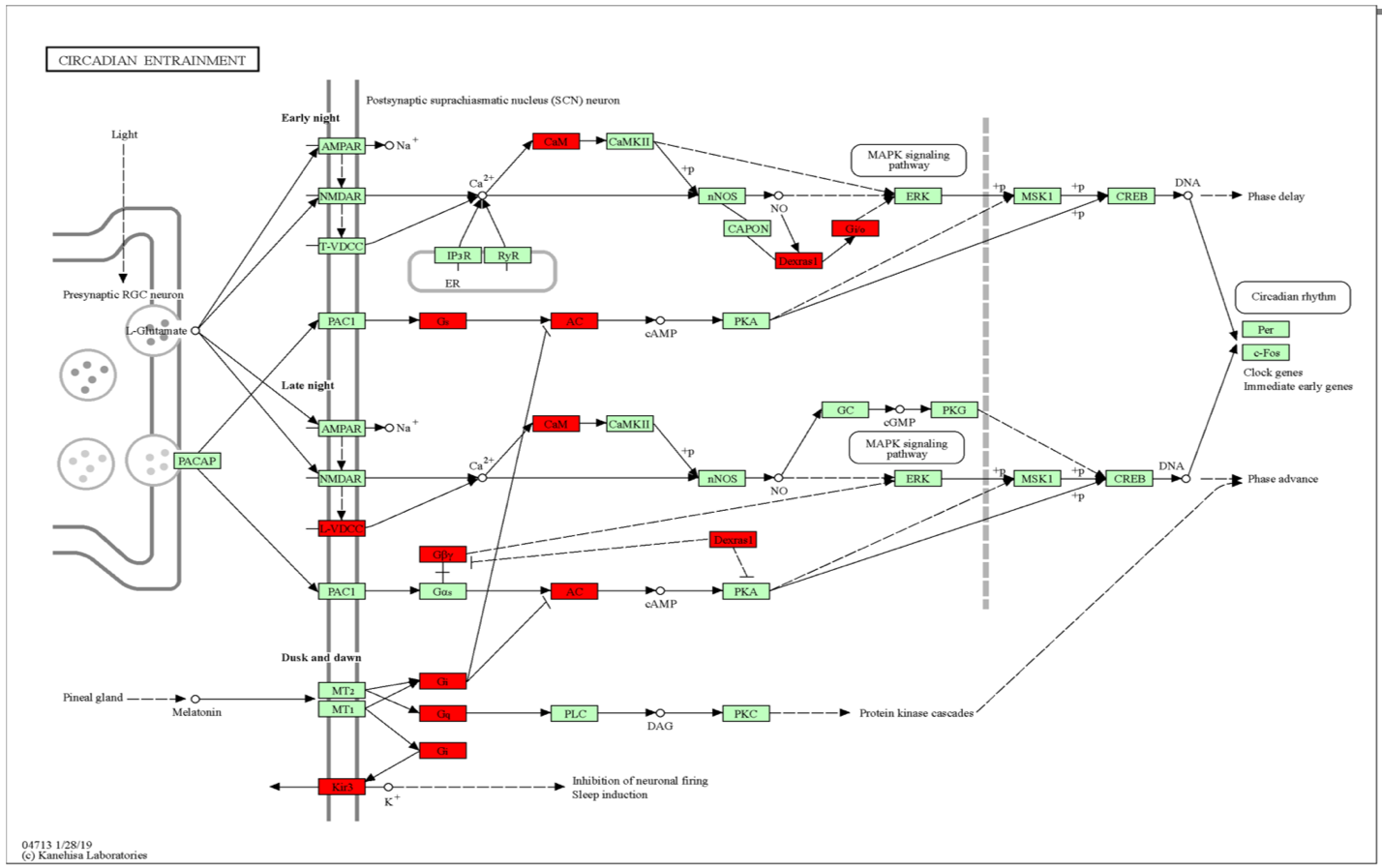
**

**Figure S1C: Genes (red) down regulated in the circadian pathways, in the pre-post-Heartfulness group**

**
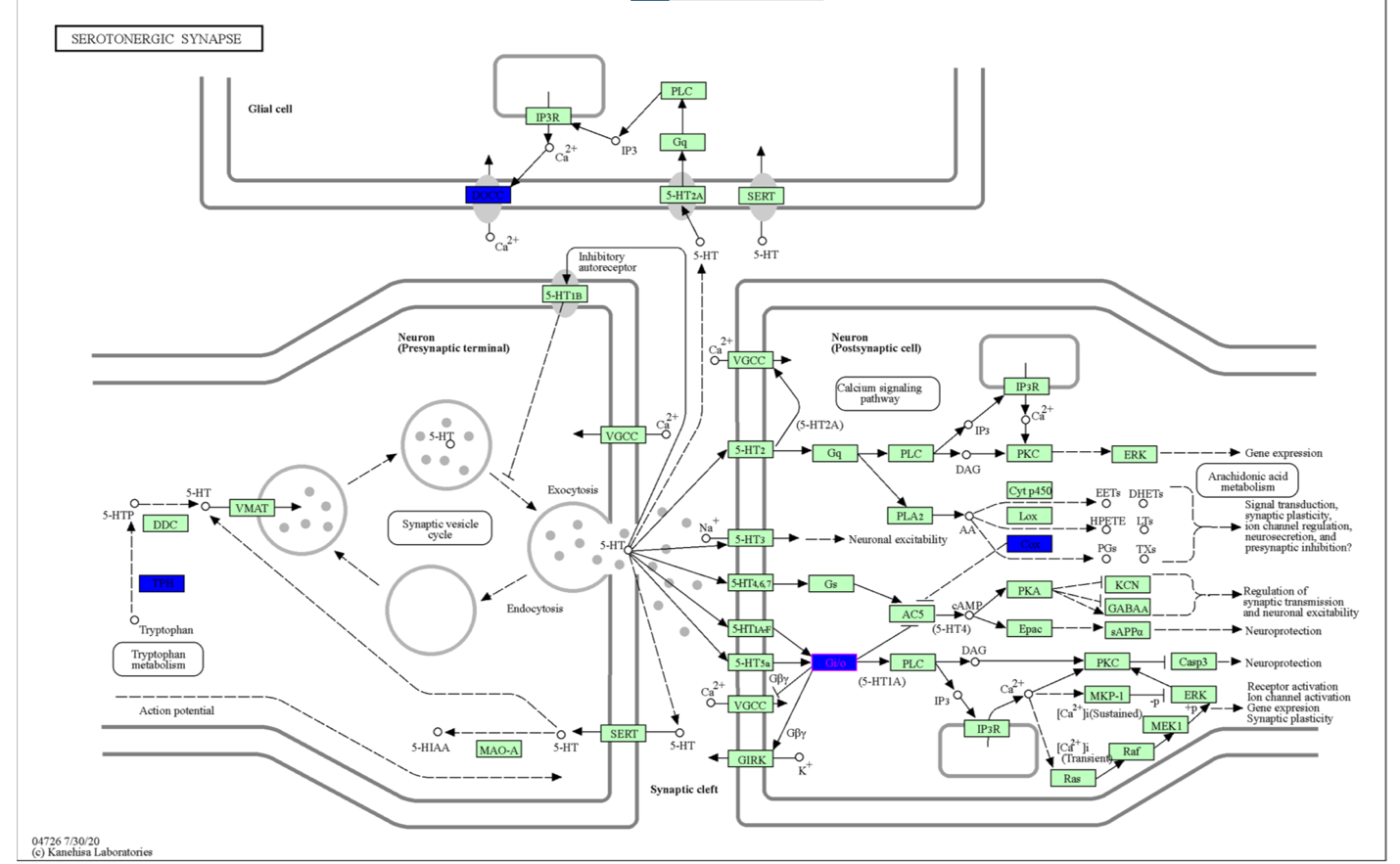
**

**Figure S1D: Genes (blue) downregulated in serotonergic process in the post-Heartfulness post-control group**

The list of genes downregulated in the comparison is shown in **Table S1.**

| Sr. No. | Gene ID | Comparison Group |
| --- | --- | --- |
| 1 | GNGT1 | Pre-post Heartfulness |
| 2 | GUCY1A2 | Pre-post Heartfulness |
| 3 | GNG12 | Pre-post Heartfulness |
| 4 | KCNJ3 | Pre-post Heartfulness |
| 5 | CALML5 | Pre-post Heartfulness |
| 6 | ADCY1 | Pre-post Heartfulness |
| 7 | ADCY6 | Pre-post Heartfulness |
| 8 | GNG13 | Pre-post Heartfulness |
| 9 | MAOA | Pre-post Heartfulness |
| 10 | NRAS | Pre-post Heartfulness |
| 11 | RASD1 | Pre-post Heartfulness |
| 12 | CACNA1D | Pre-post Heartfulness |
| 13 | PTGS2 | Post-Heartfulness post-control |
| 14 | TPH1 | Post-Heartfulness post-control |
| 15 | TRPC1 | Post-Heartfulness post-control |
| 16 | GRIN2B | Post-Heartfulness post-control |
| 17 | GNG5 | Post-Heartfulness post-control |

**Table S1.** List of genes involved in circadian entrainment pathway found to be downregulated

**Groupwise Expression Results:**

In Group 1, 615 genes were upregulated, whereas 1772 genes were downregulated. Among these, protein-coding genes accounted for 121 upregulated and 1638 downregulated genes. In Group 2, 1158 genes were upregulated, while 1989 genes were downregulated, with 206 upregulated and 1791 downregulated being protein-coding genes. Group 3 showed 721 upregulated and 1907 downregulated genes, with 90 of the upregulated and 1738 of the downregulated genes encoding proteins.

A total of 17 upregulated and 1460 downregulated protein-coding genes were common across all three groups.

Unique upregulation of coding genes was identified as follows: 84 in Group 1, 150 in Group 2, and 44 in Group 3. Additionally, unique downregulation of coding genes was observed: 59 in Group 1, 143 in Group 2, and 109 in Group 3.

KEGG pathway analysis revealed downregulation of genes associated with serotonergic signaling, apoptosis, circadian entrainment, and the IL-17 pathway in all meditation groups. Interestingly, no genes involved in these pathways were upregulated. (Details of the KEGG pathways and genes are provided in Supplementary Figures 2a-d).


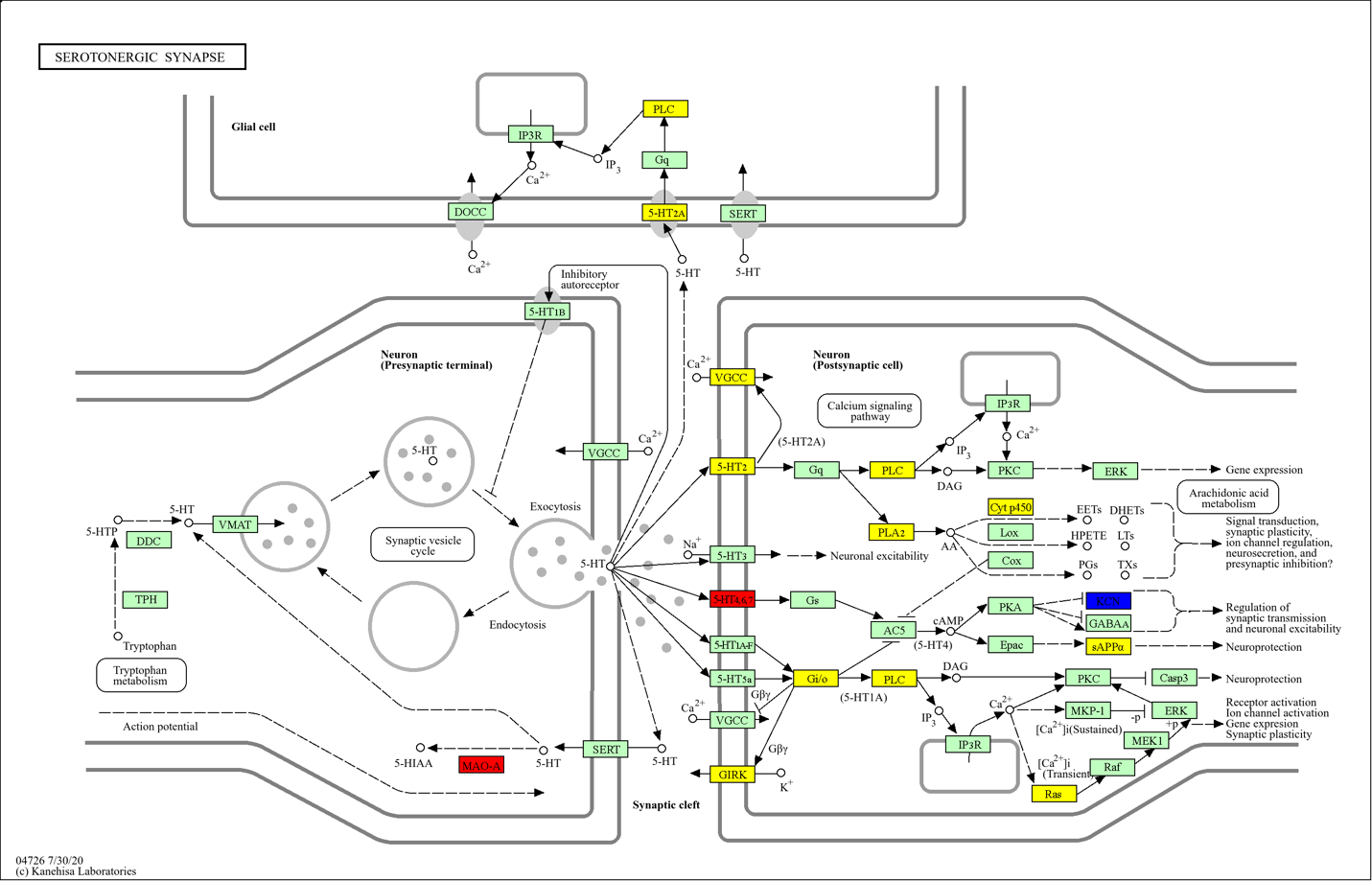


**Figure S2A: Down regulation of serotonergic pathways**

**
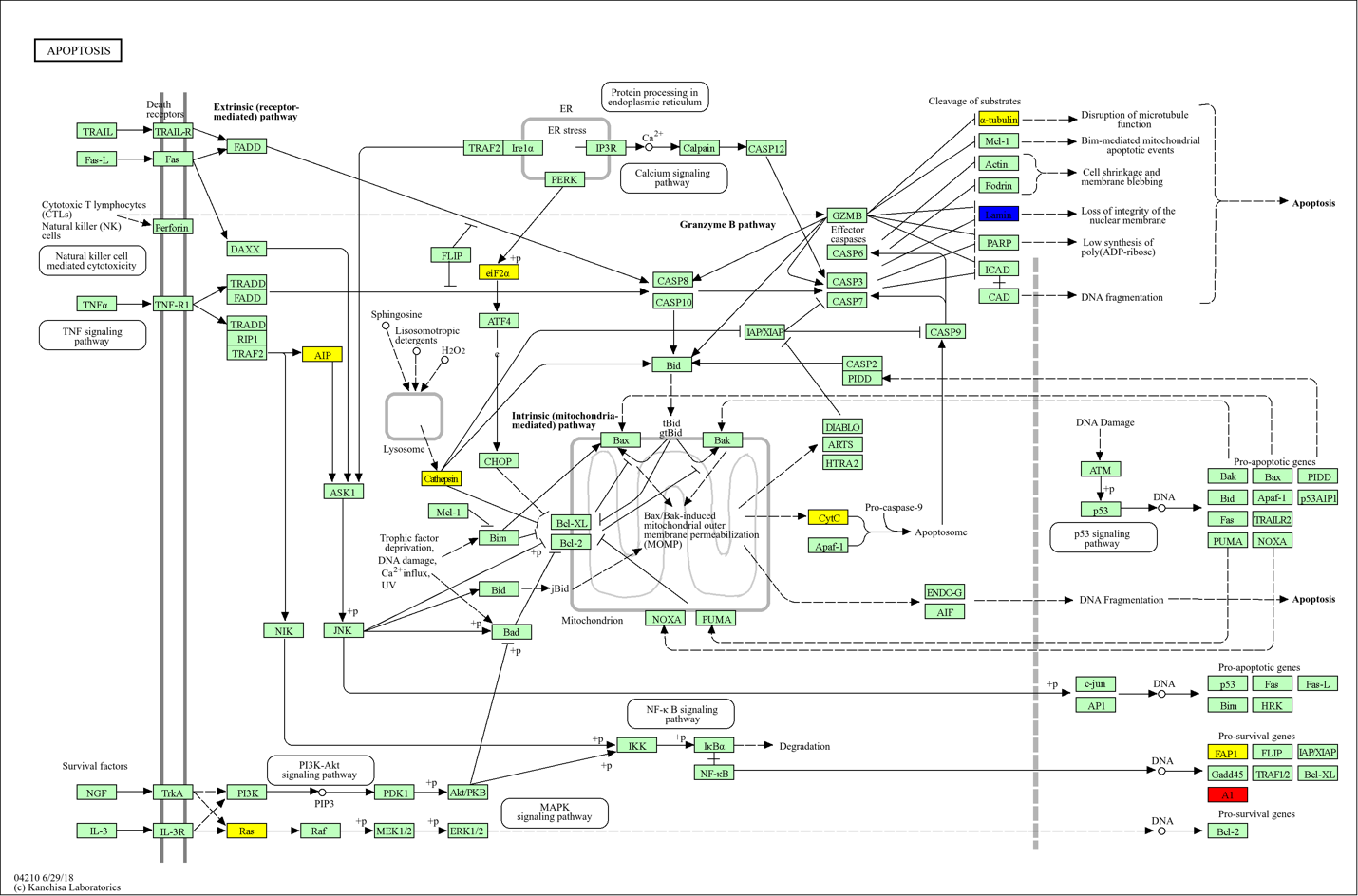
**

**Figure S2B: Down regulation of apoptotic pathways**

**
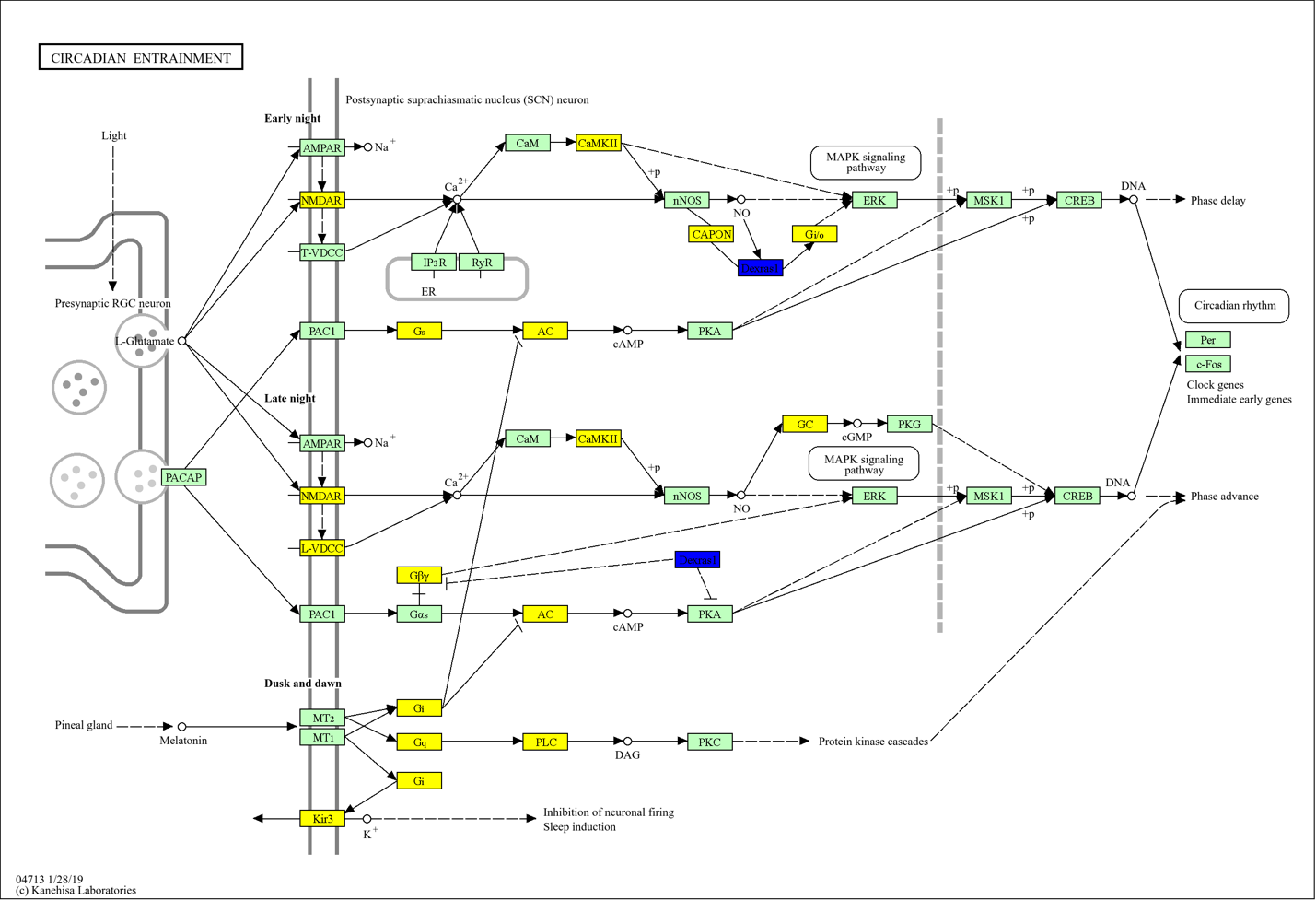
**

**Figure S2C: Down regulation of circadian entrainment pathways**

**
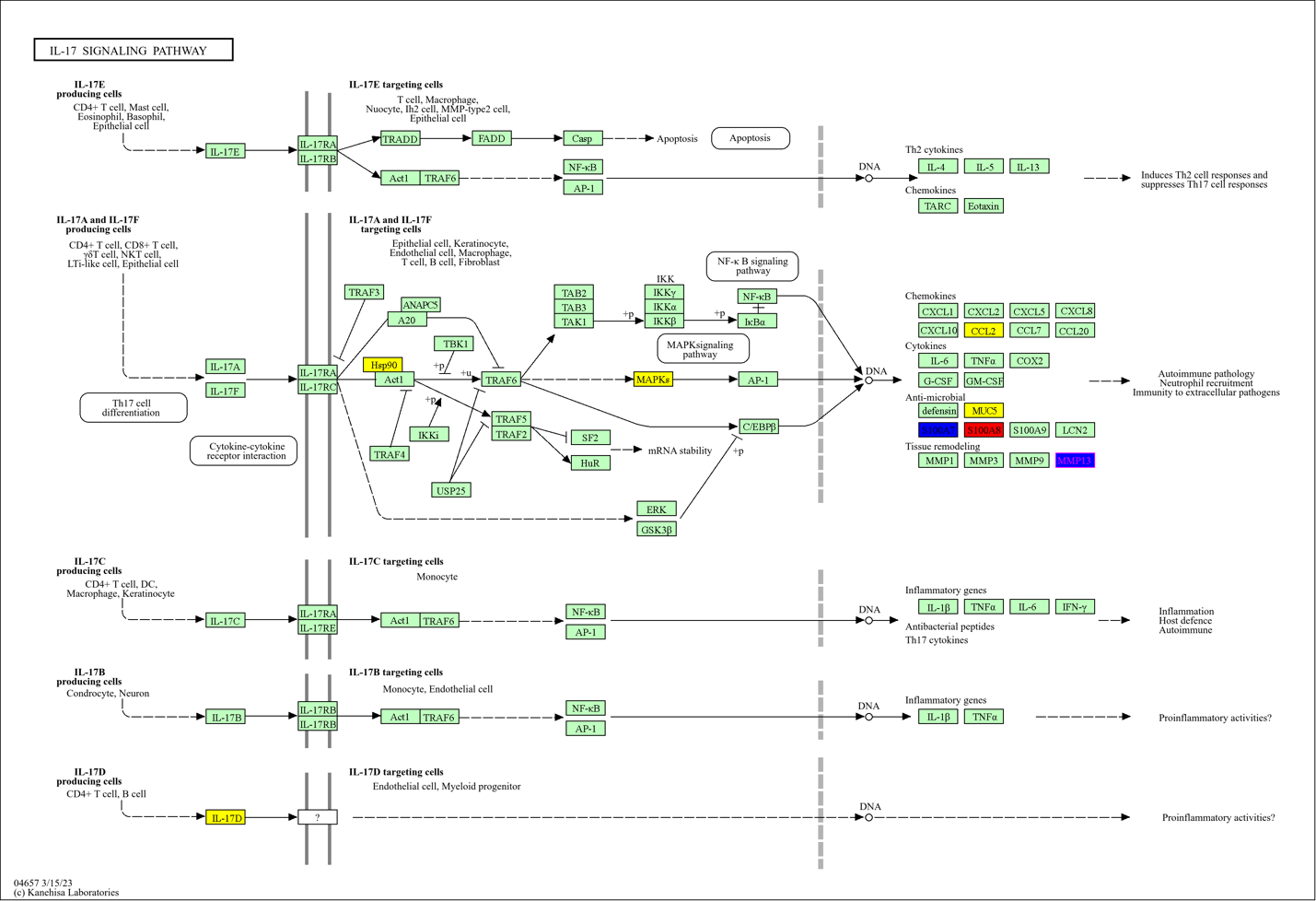
**

**Figure S2D: Down regulation of IL-17 pathways**

Group wise regulation of bioprocesses is shown in Tables S2 and S3.

| **Group1** | **Group2** | **Group3** |
| --- | --- | --- |
| IL27, MDA-5, MyD88 mediated signalling | Indolalkylamine biosynthesis,protein -pyridoxal 5 phosphate linkage, Carotene metabolic process | Protein tyrosine kinase collagen receptor activity |
| Negative regulation of IP-10 production | Spermatid nucleus elongation | Regulation of clathrin coat assembly |
| Reguation of immune response , granulocyte stimulating factor signalling | Regulation of muscle contraction and Calcium sequestering, filament sliding. | Low-affinity phosphate transmembrane transporter activity |
| Thymidylate kinase activity | Ser-tRNA(Ala) hydrolase activity | High-affinity sodium: dicarboxylate symporter activity |
| L-ascorbate:sodium symporter activity | Choline kinase activity, glycerophosphate shuttle |  |
| Radial spoke assembly, lipid ubiquitination | Interleukin-3 receptor activity |  |
|  | Cell cycle checkpoint processes, olfactory and vestibular developmental pathway |  |
|  | Broad specificity neutral L-amino acid:basic L-amino acid antiporter activity |  |
|  | Beta-alanine biosynthetic process |  |
|  | Regulation of protein linear polyubiquitination |  |
|  | Regulation of platelet formation |  |
|  | Gastric inhibitory peptide receptor activity |  |
|  | D-xylose catabolic process |  |
|  | seminal clot liquefaction |  |

**Table S2: List of Bioprocesses significantly upregulated in the group after 12 weeks of Heartfulness sessions.**

| **Group1** | **Group2** | **Group3** |
| --- | --- | --- |
| Neutral L-amino acid secondary active transmembrane transporter activity | N-acetylglucosamine biosynthetic process | Sebaceous gland cell differentiation |
| Pyridoxine biosynthetic process | ABC-type polyamine transporter activity | Vacuolar protein processing |
| Protein maturation by copper ion transfer | Protein tyrosine kinase collagen receptor activity | Glutamine secretion |
| Reelin receptor activity | Vesicle targeting, trans-Golgi to endosome | Vagus nerve morphogenesis |
| Regulation of neutrophil mediated killing of fungus | Negative regulation of ribonuclease activity | Cerebral cortex GABAergic interneuron fate commitment |
| Positive regulation of mitochondrial DNA replication | Cerebellum vasculature development | Prosaposin receptor activity |
| Modification of dendritic spine | Behavioral response to acetic acid induced pain | Regulation of synaptic transmission, glycinergic |
| Zinc ion import into synaptic vesicle | Regulation of translational initiation in response to starvation | Cellular response to vitamin E |
| Cytoplasmic translational termination | Histone H3T3 kinase activity | Positive regulation of macrophage inflammatory protein 1 alpha production |
|  | Zinc ion import into synaptic vesicle | Response to norepinephrine |
|  | Negative regulation of Fas signaling pathway | Histone H3K27 trimethyltransferase activity |
|  | Regulation of nitrosative stress-induced intrinsic apoptotic signaling pathway | Regulation of inorganic anion transmembrane transport |
|  | Ornithine biosynthetic process | Directional guidance of interneurons involved in migration from the subpallium to the cortex |
|  | Myotube cell development involved in skeletal muscle regeneration | Microvillar actin bundle assembly |
|  | Negative regulation of DNA endoreduplication | Negative regulation of brain-derived neurotrophic factor receptor signaling pathway |
|  | tRNA (guanine(37)-N(1))-methyltransferase activity | Cellular response to butyrate |
|  | translation at pre-synapse |  |
|  | Negative regulation of microvillus assembly |  |

**Table S3: List of Bioprocesses significantly downregulated in the group after 12 weeks of Heartfulness sessions.**

**GO Enrichment Analysis Results:**

**Group 1 GO Enrichment Analysis:**

The 84 uniquely upregulated genes were primarily enriched in bioprocesses involved in inflammatory processes such as IL4 and IL27 signaling regulation, MDA5 and MyD88 bioprocesses, immune regulation, thymidylate kinase activity, and lipid ubiquitination (Supplementary Figure 3a). The 59 uniquely downregulated genes were enriched in bioprocesses such as reelin receptor activity, neutral L-amino acid membrane transport activity, cytoplasmic translation termination, DNA replication, pyridoxine biosynthesis, dendritic spine modification, and neutrophil-mediated killing (Supplementary Figure 3b).


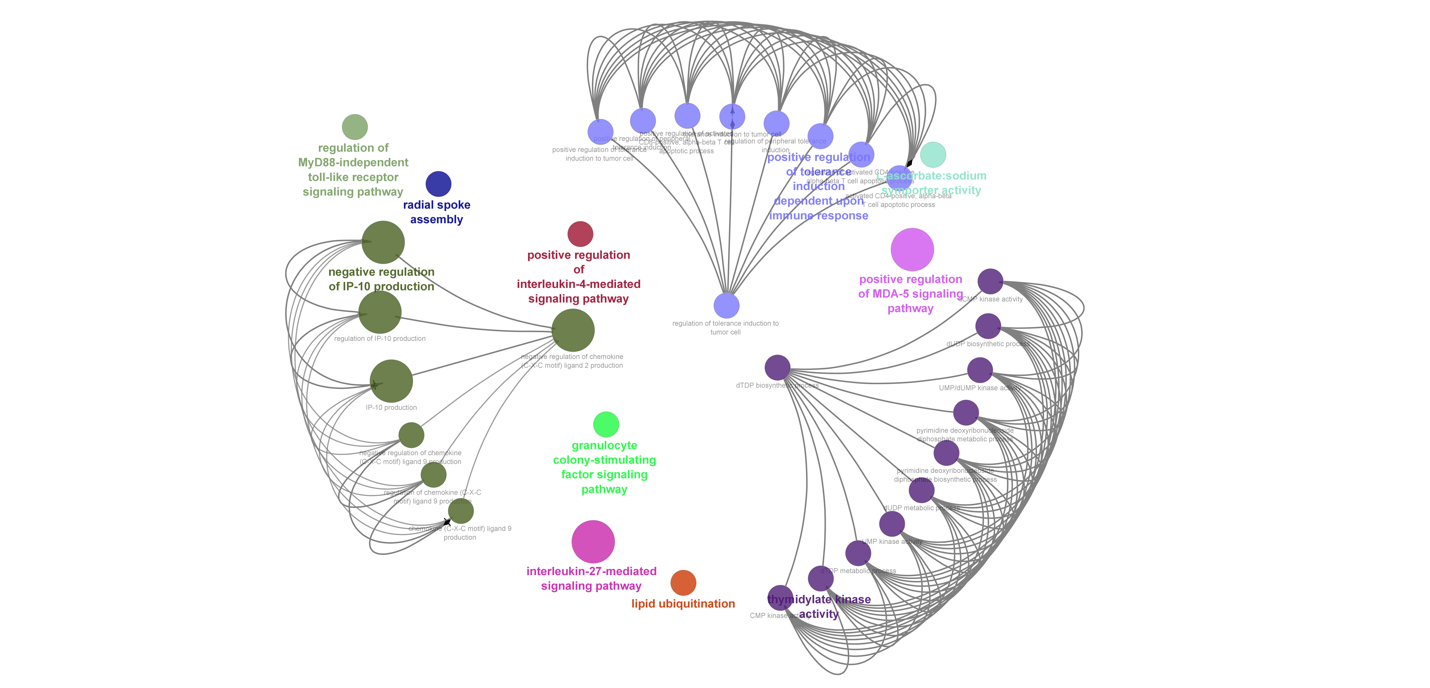


**Figure S3A: Group 1 upregulated genes**

**
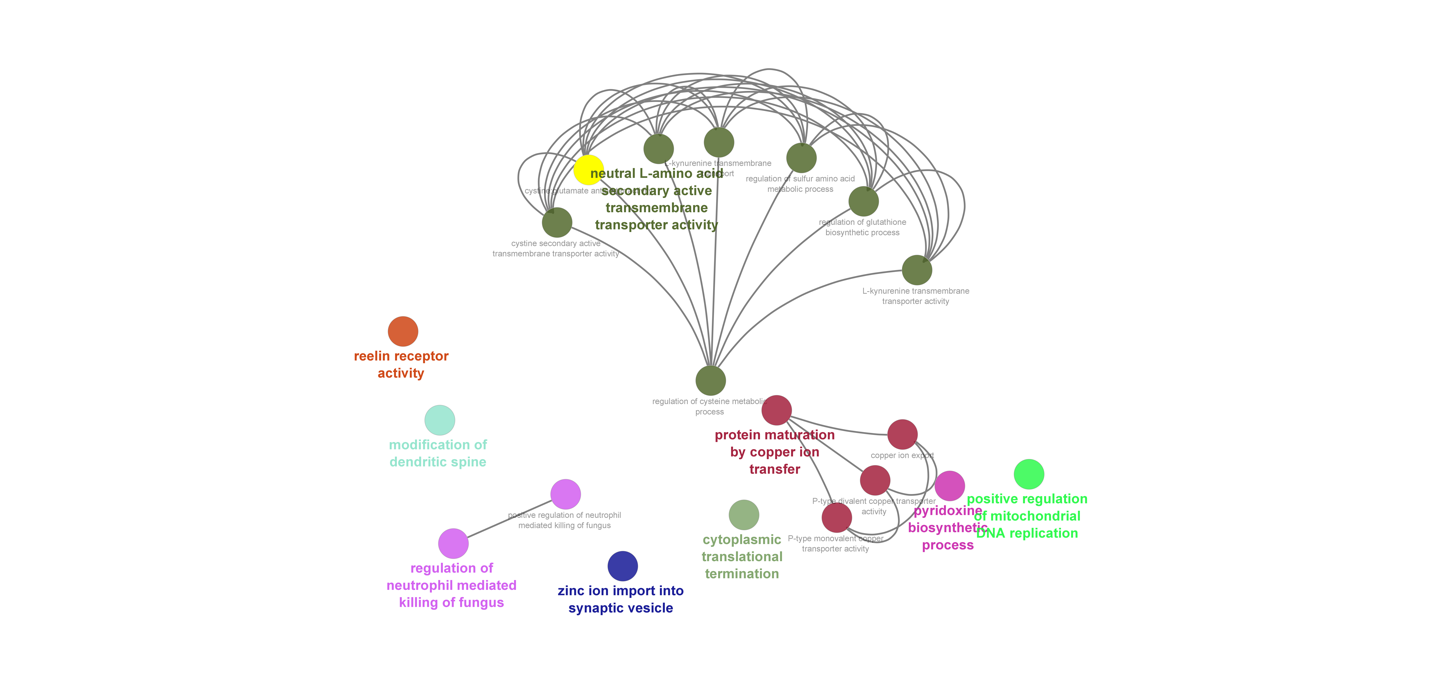
**

**Figure S3B: Group 1 downregulated genes**

**Group 2 GO Enrichment Analysis:**

The 150 uniquely upregulated genes were enriched in bioprocesses related to protein ubiquitination, carotene metabolic processes, platelet formation, spindle checkpoint, skeletal muscle contraction signaling, IL3 receptor activity, and other processes (Supplementary Figure 4a). The 143 uniquely downregulated genes were enriched in bioprocesses involving cellular responses to stress and starvation, translation regulation, histone H3T3 kinase activity, regulation of stress-induced apoptotic signaling, FAS signaling negative regulation, polyamines and N-acetylglucosamine transporter activity, and others (Supplementary Figure 4b)


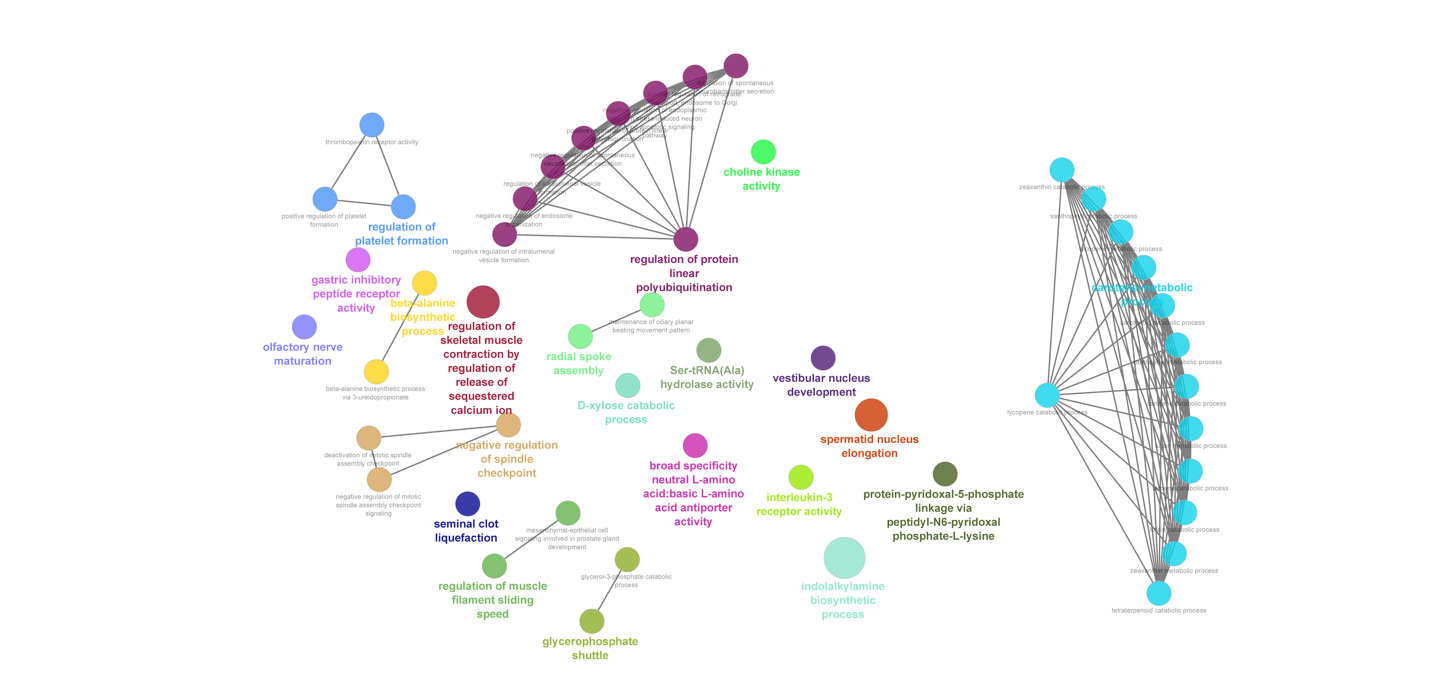


**Figure S4A: Group 2 upregulated genes**

**
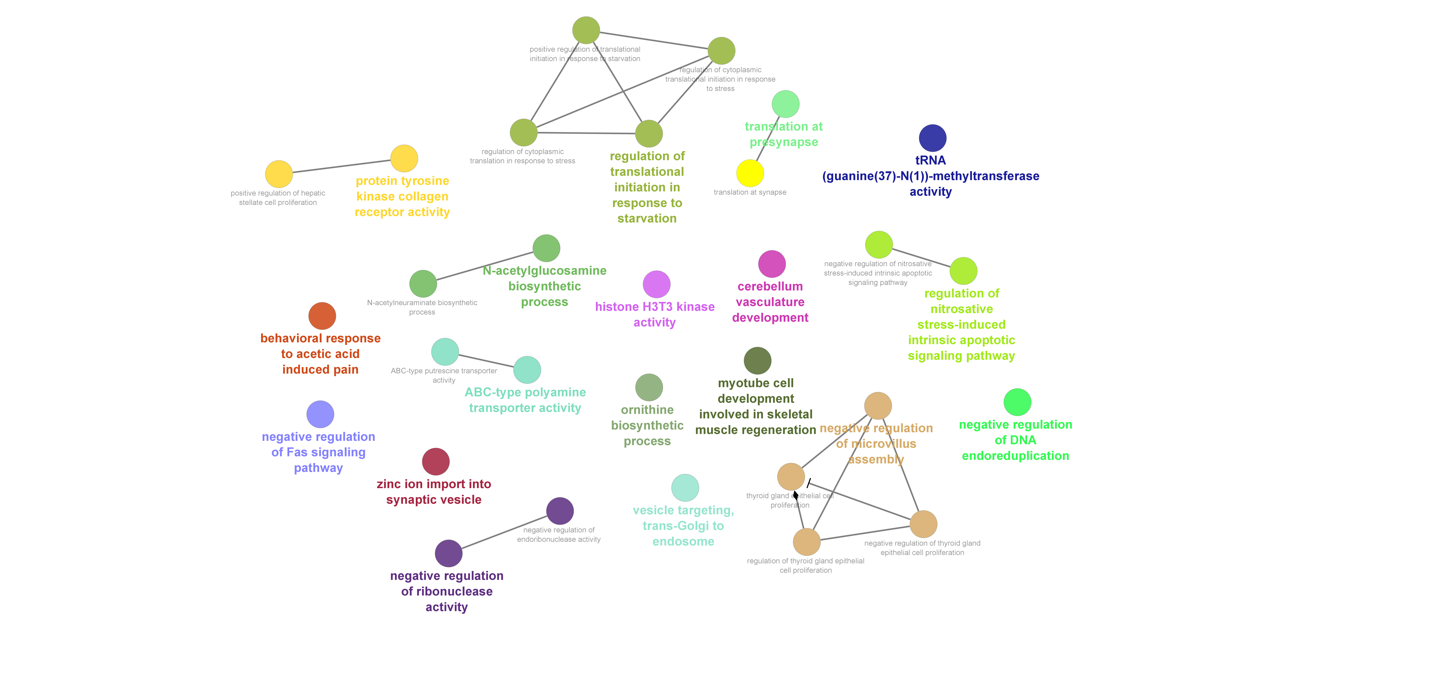
**

**Figure S4B: Group 2 downregulated genes**

**Group 3 GO Enrichment Analysis:**

The 44 uniquely upregulated genes were enriched in bioprocesses such as protein collagen receptor signaling, clathrin coat assembly, dicarboxylate-sodium symporter activity, and phosphate transmembrane transporter activity (Supplementary Figure 5a). The 109 uniquely downregulated genes were enriched in bioprocesses involved in synaptic transmission and regulation, including glutamine secretion, glycinergic synaptic signaling, brain-derived neurotrophic factor signaling, histone modification, prosaposin receptor activity, and inflammatory processes (Supplementary Figure 5b)


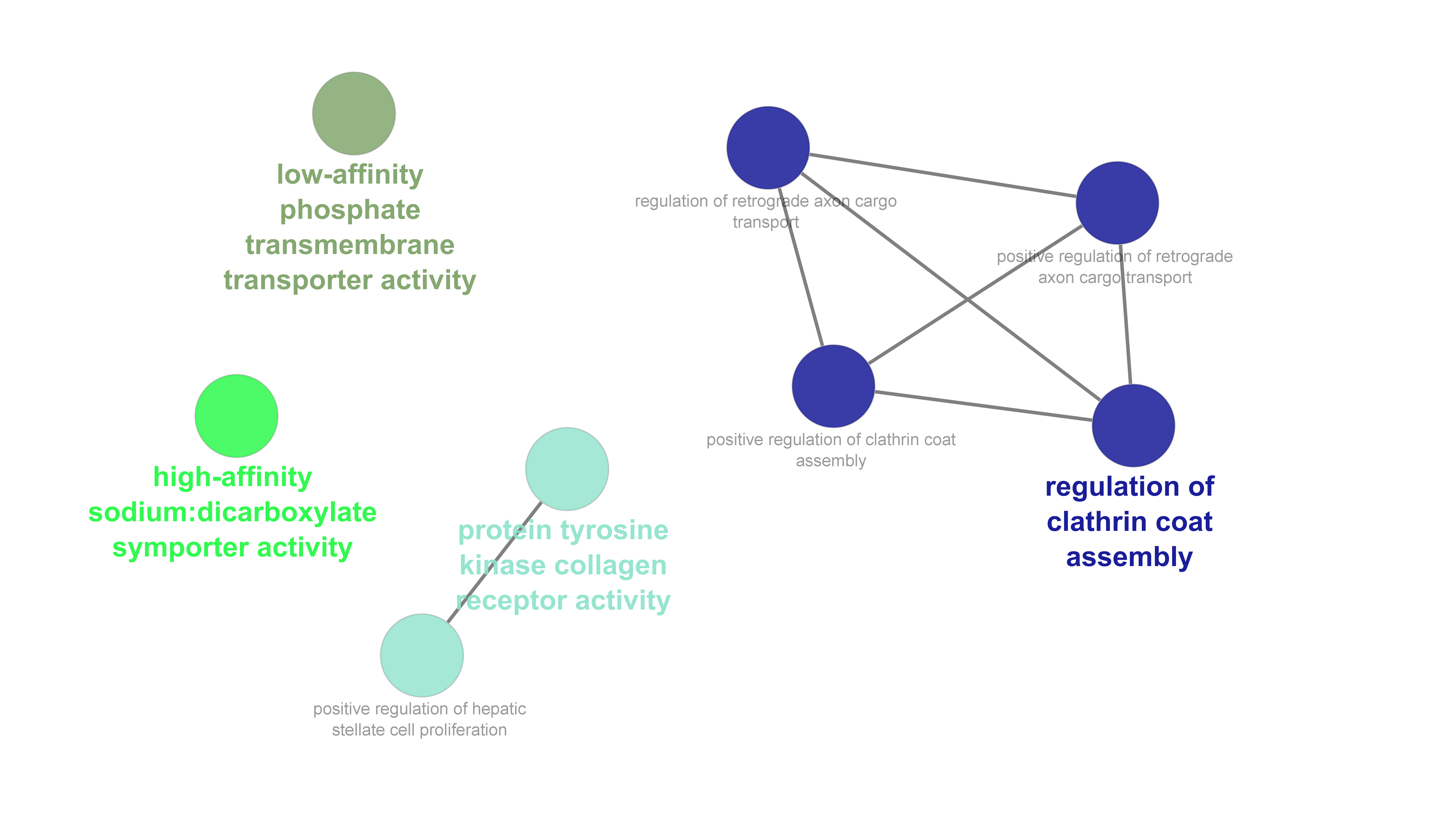


**Figure S5A: Group 3 upregulated genes**

**
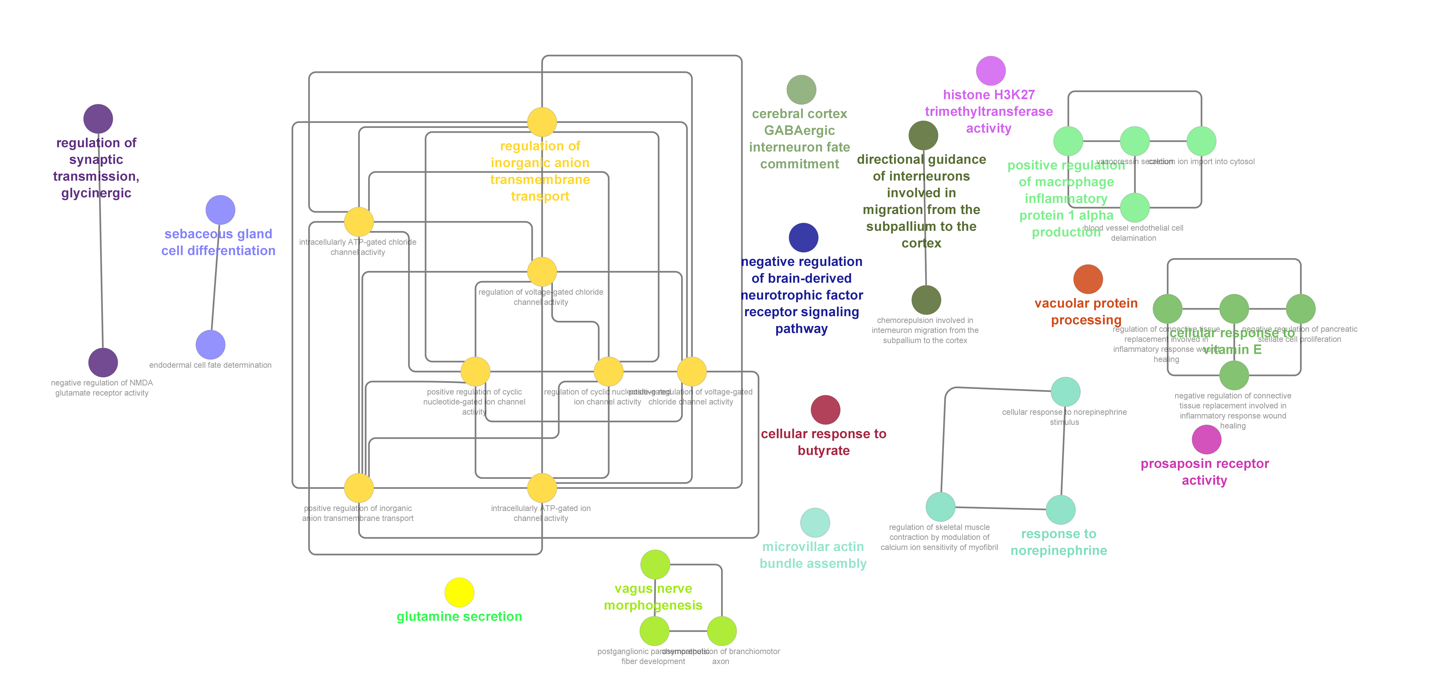
**

**Figure S5B: Group 3 downregulated genes**

**GO Enrichment Analysis of Common Protein-Coding Genes:**

The 17 commonly upregulated genes, which exhibited similar fold changes across all groups, were enriched in processes such as NAD metabolic process regulation, quinolinate and picolinate biosynthesis, meiosis regulation, and piRNA processing (Supplementary Figure 6a). The 1460 commonly downregulated genes also showed similar fold changes, except for 55 genes with a fold change greater than 1.5 log2FoldChange. These genes were enriched in bioprocesses involving DNA replication, cell cycle regulation, spindle assembly, DNA repair, developmental processes, cell polarity regulation, synaptic complexes, amyloid fibril formation, tau kinase activity, catagen processes, valine transport, and glycine betaine processes (Supplementary Figure 6b).

KEGG pathway analysis found 12 genes involved in the circadian entrainment and 9 genes of IL-17 were downregulated in all groups .


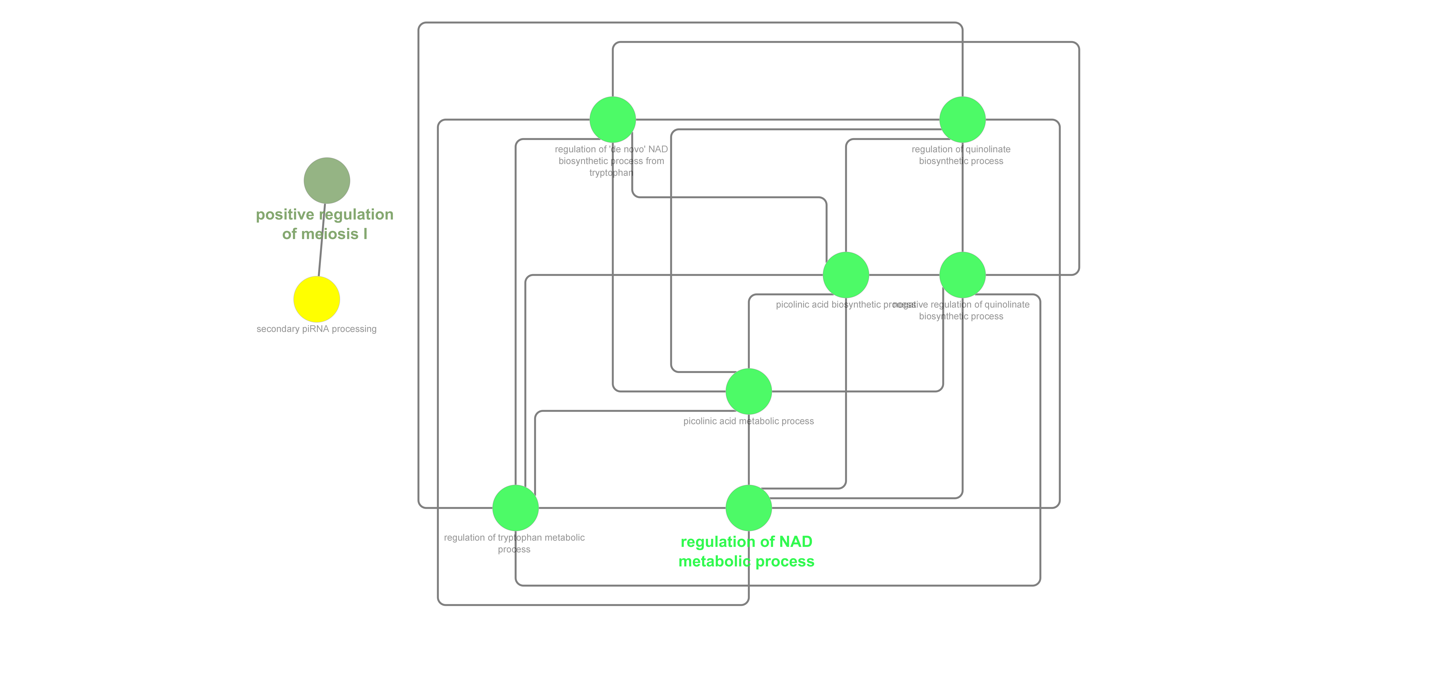


**Figure S6A: Group 1-3 common upregulated genes**

**
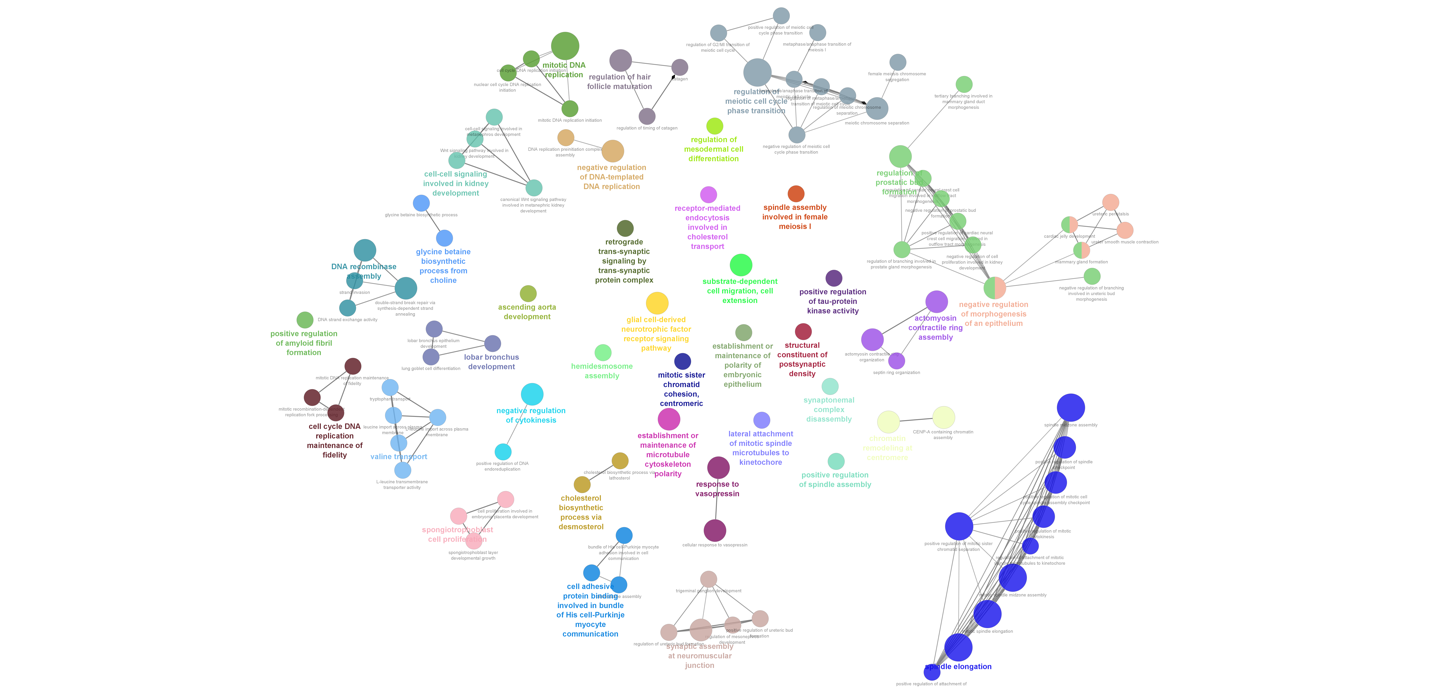
**

**Figure S6B: Group 1-3 common downregulated genes**
