# Supplementary figures and images for: Effect of a Web-Based Heartfulness Program on the Mental Well-Being, Biomarkers, and Gene Expression Profile of Health Care Students: Randomized Controlled Trial
Source: JMIR Bioinform Biotechnol. 2024 Dec 16;5:e65506. doi: 10.2196/65506 (PMC11686021; doi:10.2196/65506)

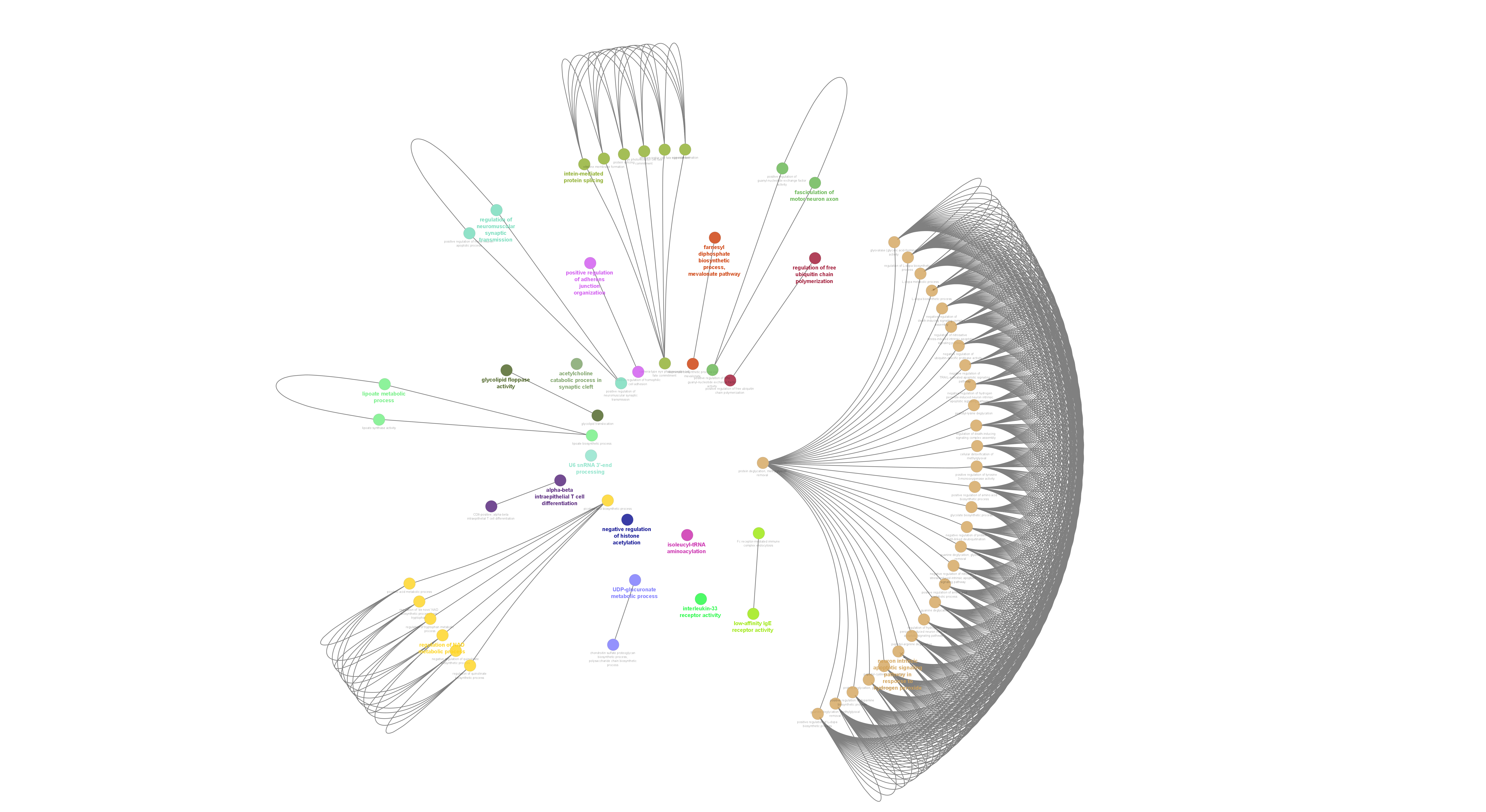

Supplement: Multimedia Appendix 3 [file bioinform_v5i1e65506_app3.png]

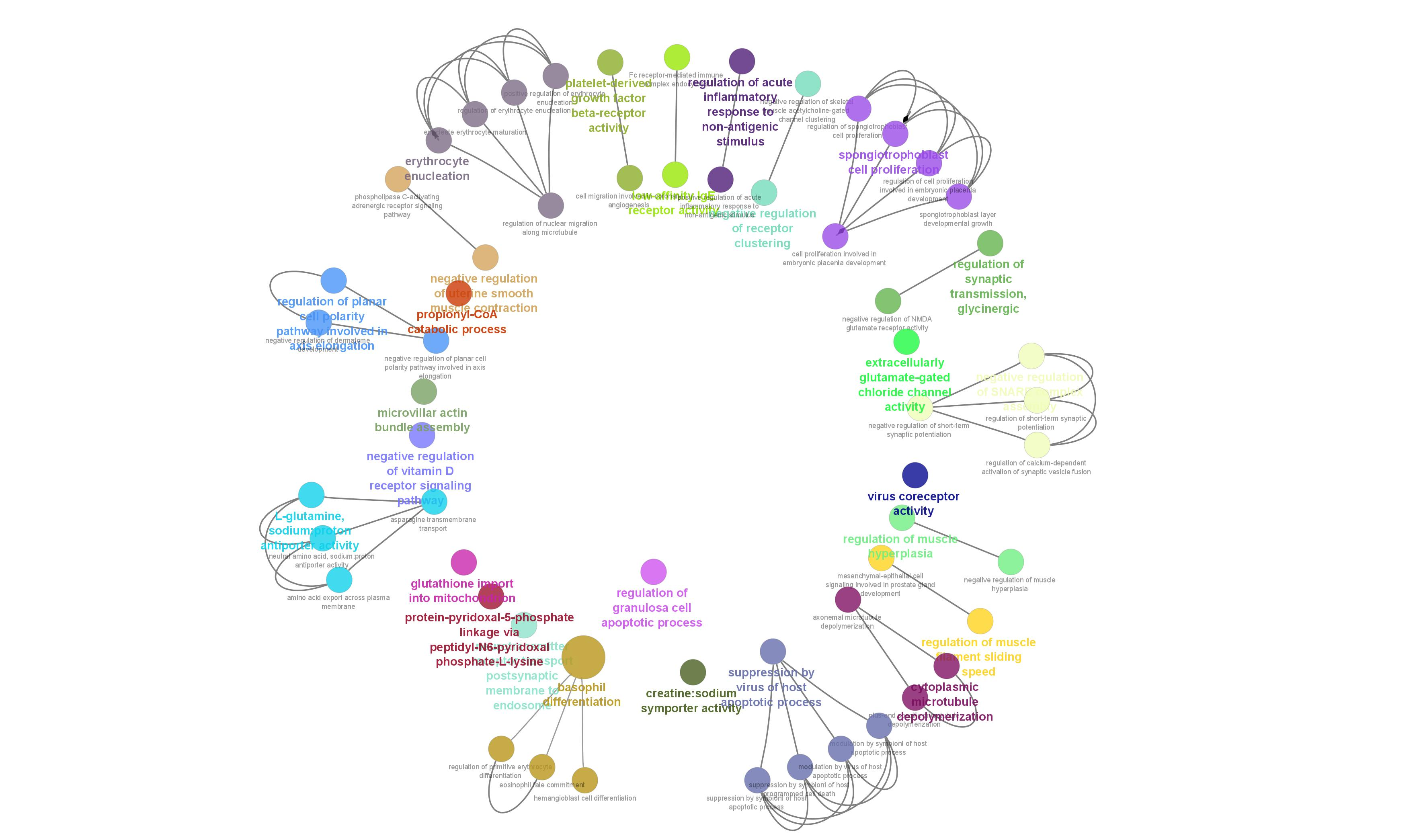

Supplement: Multimedia Appendix 4 [file bioinform_v5i1e65506_app4.png]
